# Histidinol dehydrogenase (HisD): a critical regulator of *Staphylococcus aureus* virulence and a promising target for antivirulence therapy

**DOI:** 10.1128/spectrum.01429-25

**Published:** 2025-11-26

**Authors:** Wenwen Jiang, Haotian Chen, Zejing Liu, Yingqiang Dang, Yichen Da, Zhenhua Liu, Yuyan Zhu, Lina Gao, Wenjin Yan, Jian Han, Chongge You

**Affiliations:** 1 Laboratory Medicine Center, The Second Hospital & Clinical Medical School, Lanzhou University 12426, Lanzhou, China; 2 School of Basic Medical Sciences, Lanzhou University 426140, Lanzhou, China; 3 Department of Mental Health, The Second Hospital & Clinical Medical School, Lanzhou University 12426, Lanzhou, China; Institute of Microbiology, Chinese Academy of Sciences, Beijing, China

**Keywords:** *Staphylococcus aureus*, virulence, biofilm, *hisD*, histidinol dehydrogenase, pixantrone

## Abstract

*Staphylococcus aureus* is a pathogen responsible for diverse severe infections. The global spread of methicillin-resistant *S. aureus* (MRSA) necessitates innovative therapeutic approaches beyond traditional antibiotics. *S. aureus* virulence mechanisms remain a critical concern. Targeting histidinol dehydrogenase (HisD), a key enzyme in histidine biosynthesis, presents a novel anti-virulence strategy. We used the derivative strains of Newman strains (Δ*hisD*, Δ*hisD*::pRAB-*hisD,* and WT::pRAB-*hisD*) and clinical strains to study the role of HisD in the pathogenicity of *S. aureus*. HisD inhibition by pixantrone was further evaluated. The absence of *hisD* significantly reduced hemolytic activity and biofilm formation, accompanied by the downregulation of virulence genes (*hla*, *coa*, *hlgA-C*, *lukE/S/F*, and *NWMN_1873*) and the *saeR/S* two-component system (*P* < 0.05). The expression of biofilm-inhibiting proteases was elevated, notably Aur and ScpA. Murine challenge revealed that Δ*hisD* exhibited 5.6-fold higher LD_50_ (4.17 × 10^9^ CFU/mL vs. WT 7.42 × 10^8^ CFU/mL) and reduced organ colonization (*P* < 0.05). Through structure-based virtual screening and SPR affinity verification, we discovered pixantrone—a nitrogenated anthraquinone—as a potent HisD inhibitor binding via four hydrogen bonds and two salt bridges. Pixantrone dose-dependently (25–200 μM) suppressed virulence phenotypes *in vitro*, achieving hemolysis inhibition, virulence gene inhibition, and biofilm reduction. *In vivo*, pixantrone (30 mg/kg) decreased serum CRP, IL-6, and TNF-α levels while diminishing abscess sizes and splenic bacterial loads (*P* < 0.05 vs. untreated). These findings establish HisD as a pivotal virulence regulator in *S. aureus* through *saeR/S*-mediated pathways. Pixantrone demonstrates potent anti-virulence efficacy, positioning HisD inhibition as a promising therapeutic strategy against *S. aureus* infections. This study provides foundational insights for developing HisD-targeted agents to combat antibiotic-resistant staphylococcal pathogens.

**IMPORTANCE:**

The increase in drug-resistant *Staphylococcus aureus* (MRSA) demands therapies that block virulence without promoting resistance. We identify histidinol dehydrogenase (HisD), a histidine-synthesis enzyme, as a key controller of *S. aureus* pathogenicity. Disrupting HisD genetically or with pixantrone—a newly identified inhibitor—reduces bacterial toxicity, biofilm formation, and virulence gene activity while improving survival and reducing organ damage in infected mice. Pixantrone's dose-dependent suppression of infection severity and inflammation positions it as a therapeutic candidate. Unlike traditional antibiotics, this strategy disarms bacteria rather than killing them, reducing resistance risks. By uncovering HisD’s role in connecting metabolism to virulence through the *saeR*/S system, we reveal a druggable target for fighting multidrug-resistant infections. This work addresses the urgent need for innovative solutions to the global antibiotic resistance crisis, paving the way for therapies that outsmart evolving superbugs.

## INTRODUCTION

*Staphylococcus aureus*, a zoonotic gram-positive pathogen, is a leading cause of skin infections, pneumonia, sepsis, and toxin-mediated syndromes such as life-threatening toxic shock. As the primary etiological agent of bloodstream infections, *S. aureus* accounts for 15%–30% of fatal bacteremia cases, contributing to approximately 300,000 annual deaths globally (1). First-line β-lactam antibiotics (e.g., methicillin) target cell wall biosynthesis; however, the increase in methicillin-resistant *S. aureus* (MRSA)—responsible for ~120,000 deaths annually (2)—and vancomycin-intermediate *S. aureus* (VISA) has prompted the World Health Organization (WHO) to classify these strains as critical-priority superbugs (3). This antimicrobial resistance crisis has intensified interest in anti-virulence therapy, a strategy that attenuates pathogenicity by disrupting virulence factor production rather than directly killing pathogens, thereby enabling host immune clearance (4, 5).

A mechanistic understanding of *S. aureus* virulence determinants is pivotal for advancing such therapies. The pathogen’s virulence arsenal includes cytotoxins (e.g., α-toxin, Panton-Valentine leukocidin [PVL/LukSF-PV], HlgAB/CB, and LukED), superantigens (e.g., staphylococcal enterotoxins, toxic shock syndrome toxin-1), and lytic enzymes (e.g., β-hemolysin, exfoliative toxins) (6, 7). Additionally, *S. aureus* employs tissue-degrading enzymes (coagulase, staphylokinase, and proteases) and biofilm formation to evade host defenses and establish infection (8). These virulence mechanisms are tightly regulated by interconnected systems, including the Agr quorum-sensing network, the SaeRS two-component system, SarA-family proteins, and SigB (9). These virulence factors and regulatory systems help bacteria tolerate and escape immune clearance of the body during infection, creating an environment for bacteria to multiply and cause disease.

Histidinol dehydrogenase (HisD), a Zn²^+^-dependent enzyme absent in mammals, catalyzes the final two NAD^+^-dependent oxidative steps in L-histidine biosynthesis (10–13). Its conservation across bacteria and plants (14) and essentiality in pathogens like *Mycobacterium tuberculosis* and *Brucella suis* (12, 15–18) position HisD as an attractive antimicrobial target. HisD-deficient *Salmonella* and *Micrococcus luteus* exhibit impaired colonization and attenuated virulence (19, 20), whereas phylogenetic analyses reveal structural homology between bacterial HisD and yeast HIS4C, underscoring its evolutionary significance.

Despite these advances, the role of HisD in *S. aureus* pathogenesis remains unexplored. Here, we investigate HisD’s regulatory impact on *S. aureus* virulence factor production and biofilm formation. Using genetic knockout (Δ*hisD*), complemented (Δ*hisD*::pRAB-*hisD*), and overexpression (WT::pRAB-*hisD*) strains, coupled with the HisD inhibitor pixantrone (PIX), we demonstrate that HisD critically modulates hemolytic activity, biofilm dynamics, and murine pathogenicity. Our findings reveal that pixantrone attenuates *S. aureus* virulence *in vitro* and *in vivo*, highlighting HisD as a novel target for anti-virulence therapeutics.

## RESULTS

### *hisD* deletion attenuates hemolytic activity in *S. aureus*

**Fig 1:**
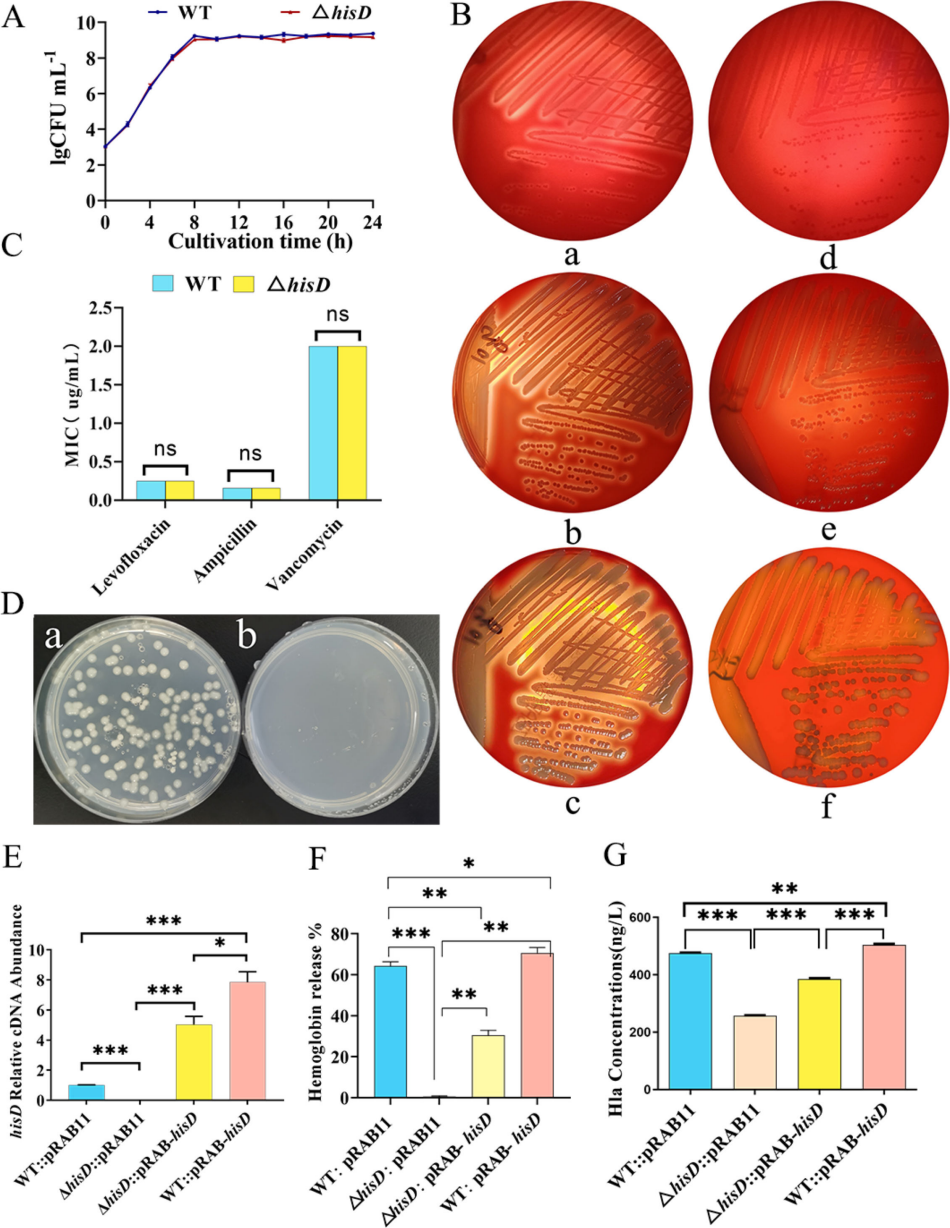
Impact of *hisD* deletion on growth, antibiotic susceptibility, and hemolytic activity in *S. aureus*. (**A**) Growth curves of wild-type (WT) and Δ*hisD* strains during logarithmic and stationary phases in TSB. (**B**) Hemolytic activity on sheep blood agar: (Ba–Bc) WT colonies at 12, 24, and 36 h; (Bd–Bf) Δ*hisD* colonies at corresponding time points. (**C**) Minimal inhibitory concentrations (MICs) of ampicillin (β-lactam), levofloxacin (fluoroquinolone), and vancomycin (glycopeptide). (**D**) The Δ*hisD* strain formed colonies on histidine-supplemented basal medium (a) but showed no growth in its absence (b). (**E**) *hisD* transcript levels in the indicated strains: WT::pRAB11 (empty vector control), Δ*hisD*::pRAB11 (knockout with vector), Δ*hisD*::pRAB-*hisD* (complemented strain), and WT::pRAB-*hisD* (*hisD*-overexpressing strain). (**F**) Hemolysis rates across strains. (**G**) α-Hemolysin (Hla) levels quantified by ELISA. Significance: *P < 0.05, **P < 0.01, ***P < 0.001, ns *P* > 0.05.

To elucidate the role of *hisD* in *S. aureus* pathogenicity, we constructed a *hisD* knockout strain (Δ*hisD*) via homologous recombination. Growth kinetics analysis revealed comparable bacterial counts between wild-type (WT) and Δ*hisD* strains during both logarithmic and stationary phases (Fig. 1A). Consistently, antibiotic susceptibility assays demonstrated identical minimal inhibitory concentrations (MICs) for ampicillin (β-lactam), levofloxacin (fluoroquinolone), and vancomycin (glycopeptide) in WT and Δ*hisD* (Fig. 1C), confirming that *hisD* deletion does not alter fundamental growth dynamics or intrinsic antibiotic resistance. The Δ*hisD* strain formed colonies on histidine-supplemented basal medium but showed no growth in its absence (Fig. 1D), confirming its histidine auxotrophy. Strikingly, Δ*hisD* exhibited profoundly attenuated hemolytic activity. On sheep blood agar, Δ*hisD* colonies formed narrower hemolytic zones with delayed expansion compared with WT at 12, 24, and 36 h post-inoculation (Fig. 1B). To establish causality, we engineered complementation (Δ*hisD*::pRAB-*hisD*), *hisD*-overexpression (WT::pRAB-*hisD*), and vector control strains (WT::pRAB11, Δ*hisD*::pRAB11). *hisD* transcript levels in the four strains are shown in Fig. 1E. *hisD* expression was undetectable in Δ*hisD*::pRAB11. Compared with WT::pRAB11, *hisD* transcript levels were 5-fold higher in Δ*hisD*::pRAB-*hisD* and 7.8-fold higher in WT::pRAB-*hisD* (*P* < 0.05). Hemolysis rates and α-hemolysin levels in Δ*hisD*::pRAB11 were reduced relative to WT::pRAB11 (*P <* 0.05). Genetic complementation partially restored these two parameters to the WT level, whereas *hisD* overexpression increased hemolysis and the release of α-hemolysin (*P* < 0.05) (Fig. 1F and G).

### *hisD* as a critical modulator of *S. aureus* virulence factor production and the regulatory SaeRS two-component system

**Fig 2:**
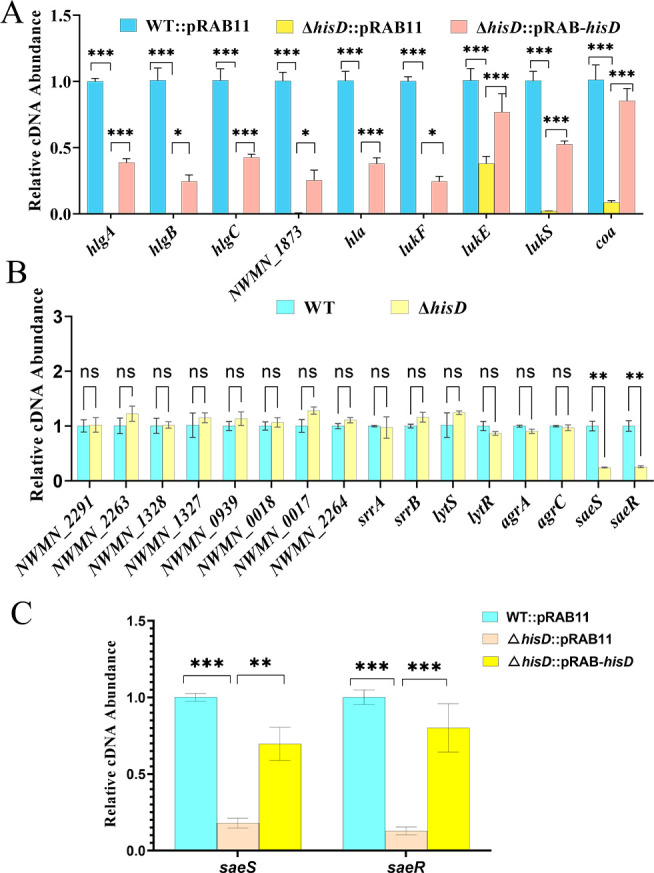
Impact of *hisD* deletion on virulence gene and two-component system (TCS) regulator expression. (**A**) *hisD*-dependent expression of virulence genes (*hla, coa, NWMN_1873, hlgA, hlgB, hlgC, lukE, lukS, lukF*) in WT::pRAB11, Δ*hisD*::pRAB11, and Δ*hisD*::pRAB-*hisD* strains. (**B**) Expression of TCS regulator genes in WT versus Δ*hisD*. TCS genes analyzed: Agr (*agrA/agrC*), Sae (*saeR/saeS*), Arl (*NWMN_1327*/*NWMN_1328*), LytSR (*lytS/lytR*), WalkR (*NWMN_0017*/*NWMN_0018*), SrrAB (*srrA/srrB*), AirSR (*NWMN_0939*), NreBC (*NWMN_2291*), and HssRS (*NWMN_2263*/*NWMN_2264*). (**C**) *saeR/saeS* expression in WT::pRAB11, Δ*hisD*::pRAB11, and Δ*hisD*::pRAB-*hisD* strains. Significance: *P < 0.05, **P < 0.01, ***P < 0.001; ns *P* > 0.05).

To elucidate the molecular basis of these phenotypic changes, we quantified mRNA levels of virulence-associated genes (*hla*, *coa*, *NWMN_1873*, *hlgA*, *hlgB*, *hlgC*, *lukE*, *lukS*, *lukF*) and genes encoding two-component regulatory systems (TCS): *agrA/agrC* (Agr), *saeR/saeS* (Sae), *NWMN_1327*/*NWMN_1328* (Arl), *lytS/lytR* (LytSR), *NWMN_0017*/*NWMN_0018* (WalkR), *srrA/srrB* (SrrAB), *NWMN_0939* (AirSR), *NWMN_2291* (NreBC), and *NWMN_2263*/*NWMN_2264* (HssRS) via qRT-PCR. Compared with WT::pRAB11, Δ*hisD*::pRAB11 exhibited significant downregulation of all virulence genes tested (*P* < 0.05), with partial transcriptional restoration in the complemented strain (Fig. 2A). Among TCS genes, *saeR* and *saeS* expression was significantly reduced in Δ*hisD*::pRAB11 versus WT::pRAB11 (*P* < 0.05), whereas other TCS genes remained unchanged (Fig. 2B). Complementation of *hisD* rescued *saeR/S* expression (Fig. 2C). These data establish HisD as a critical modulator of *S. aureus* virulence factor production and the SaeRS TCS pathway.

### *hisD* deletion abolishes biofilm formation in *S. aureus*

**Fig 3:**
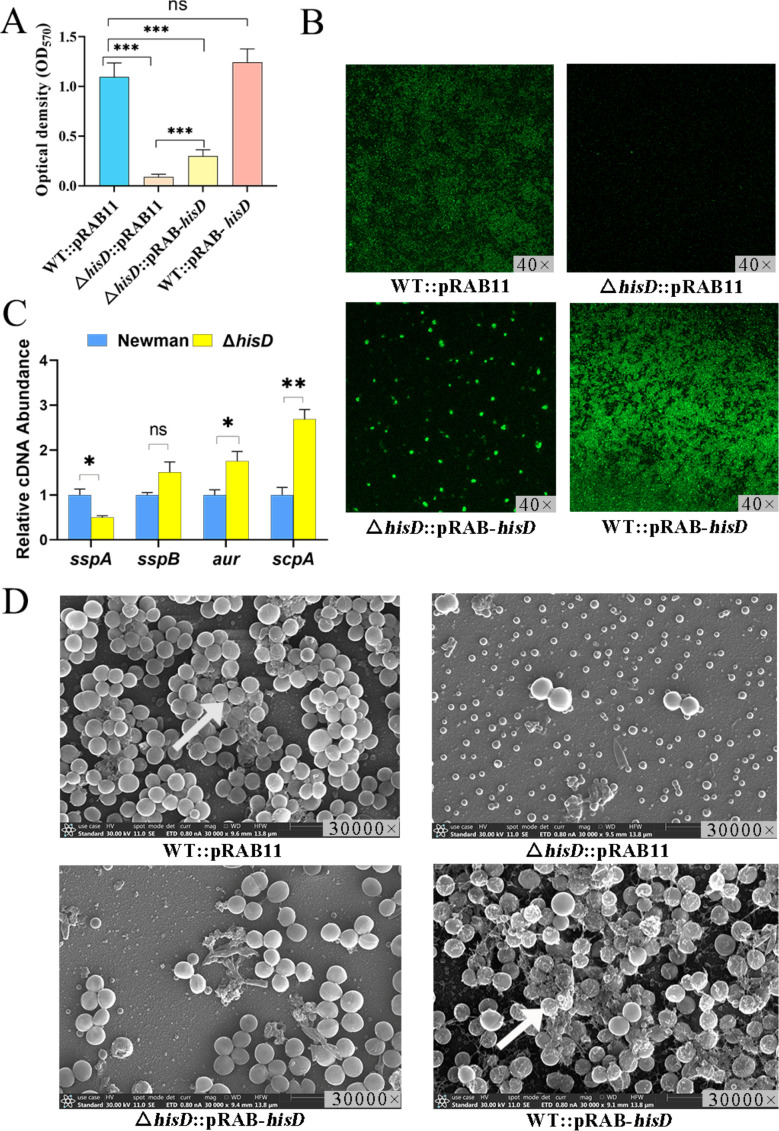
*hisD* deletion impairs biofilm formation in *S. aureus*. (**A**) Biofilm biomass quantification via crystal violet staining (OD_570nm_). Strains: WT::pRAB11, Δ*hisD*::pRAB11, Δ*hisD*::pRAB-*hisD*, and WT::pRAB-*hisD*. ***P < 0.001, ns *P* > 0.05. (**B**) Laser scanning confocal microscopy (LSCM) images of biofilms stained for polysaccharides (green fluorescence). WT::pRAB11 biofilms exhibited dense polysaccharide matrices, whereas Δ*hisD*::pRAB11 showed sparse fluorescence. Polysaccharide deposition was partially restored in Δ*hisD*::pRAB-*hisD* and enhanced in WT::pRAB-*hisD*. (**C**) qRT-PCR analysis of *sspA*, *sspB*, *aur*, and *scpA* expression in WT and Δ*hisD* strains. *P < 0.05, **P < 0.01, ns *P* > 0.05). (**D**) Scanning electron microscopy (SEM) analysis. WT::pRAB11 formed robust biofilms with abundant extracellular polysaccharide (EPS, arrows). Δ*hisD*::pRAB11 displayed sparse cell aggregation with minimal EPS. Complementation (Δ*hisD*::pRAB-*hisD*) restored cellular density, whereas overexpression (WT::pRAB-*hisD*) increased EPS production (arrows).

We assessed biofilm formation in *S. aureus* strains (WT::pRAB11, Δ*hisD*::pRAB11, Δ*hisD*::pRAB-*hisD*, and WT::pRAB-*hisD*) using crystal violet quantification, laser scanning confocal microscopy (LSCM), and scanning electron microscopy (SEM). Genetic ablation of *hisD* completely abrogated biofilm formation, as evidenced by a reduction in crystal violet absorbance compared with WT::pRAB11 (*P* < 0.05) (Fig. 3A). LSCM and SEM imaging revealed sparse, disorganized cellular aggregates in Δ*hisD*::pRAB11, contrasting with the dense, structured biofilms of WT::pRAB11 (Fig. 3B and D). Δ*hisD*::pRAB-*hisD* restored biofilm biomass to 28% of WT levels (*P* < 0.05 vs.WT::pRAB11), whereas Δ*hisD*::pRAB-*hisD* enhanced biofilm formation by 3.32-fold relative to Δ*hisD*::pRAB11 (*P* < 0.05) (Fig. 3A). Microscopic analyses confirmed these trends: Δ*hisD*::pRAB-*hisD* exhibited intermediate biofilm architecture, whereas WT::pRAB-*hisD* displayed hyperaggregation with thickened extracellular matrix deposition (Fig. 3B and D). qRT-PCR analysis revealed that the protease-encoding genes *aur* and *scpA* were significantly overexpressed (*P* < 0.05) in the Δ*hisD* strain compared with WT, and sspA was downregulated (*P* < 0.05) (Fig. 3C).

### *hisD* deficiency attenuates *S. aureus* virulence in a murine infection model

**Fig 4:**
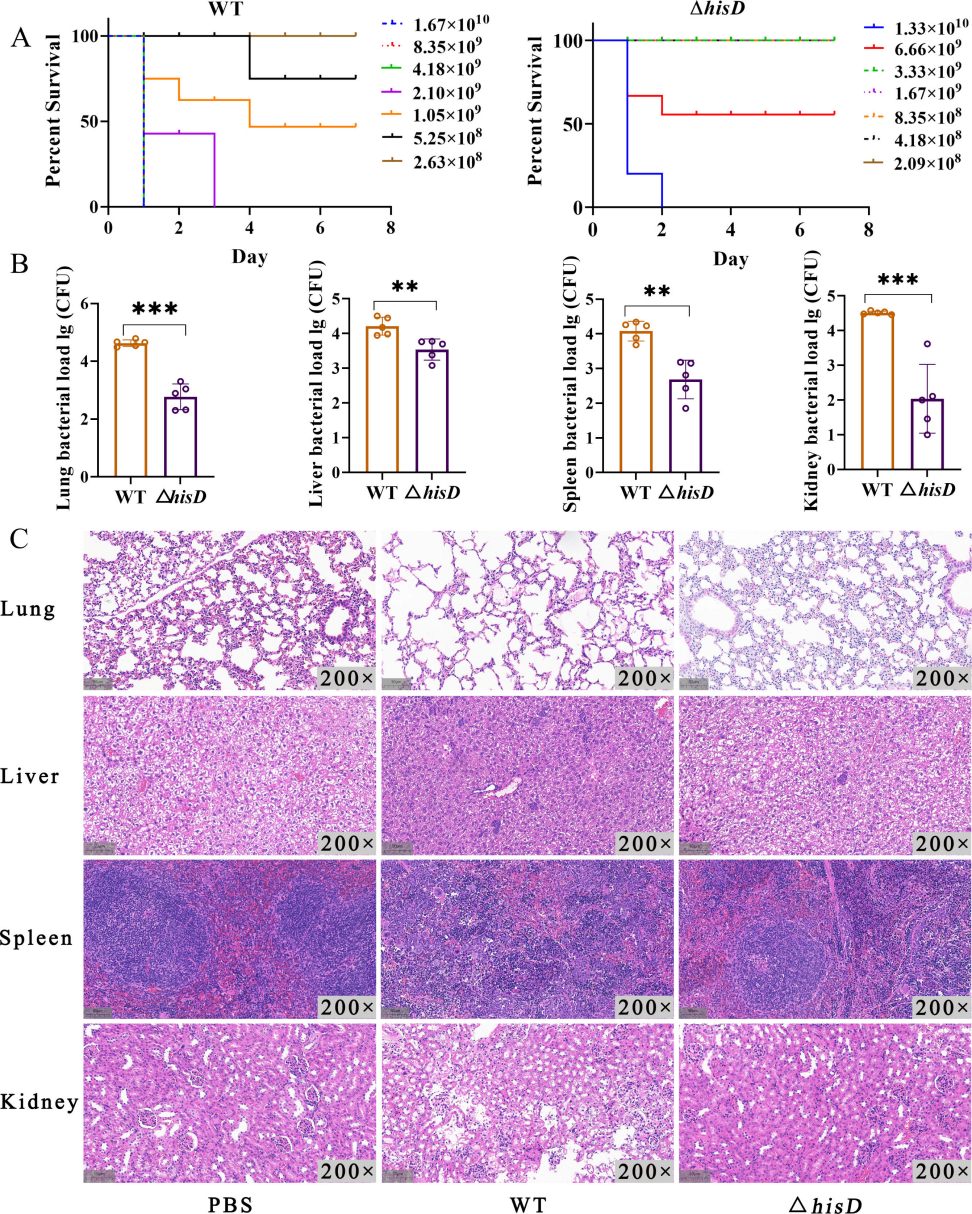
*hisD* deletion attenuates *S. aureus* virulence in BALB/c mice. (**A**) Survival curves of mice intraperitoneally challenged with escalating doses of wild-type (WT) or Δ*hisD* strains (*n* = 5 per group). LD_50_: WT = 7.42 × 10^8^ CFU/mL; Δ*hisD* = 4.17 × 10^9^ CFU/mL. (**B**) Bacterial burdens in liver, spleen, kidneys, and lungs at 7 days post-infection (1.5 × 10^8^ CFU/mL, 600 µL) (*n* = 5 per group). Data represent mean ± SD; **P < 0.01, ***P < 0.001 *vs. WT.* (**C**) Representative H&E-stained sections of lung, liver, spleen, and kidney tissues from PBS-treated, WT-infected, and Δ*hisD*-infected mice (*n* = 3 per group). Lung: PBS—intact alveolar architecture; WT—severe neutrophilic infiltration, alveolar collapse, and capillary deformation; Δ*hisD*—mild alveolar wall thickening and focal inflammation. Liver: PBS—normal hepatocyte arrangement; WT—focal inflammatory aggregates; Δ*hisD*—reduced leukocyte infiltration. Spleen: PBS—distinct white pulp and red pulp; WT—effaced lymphoid follicles and diffuse inflammation; Δ*hisD*—partial white/red pulp demarcation with residual follicles. Kidney: PBS—organized glomeruli and tubules; WT—glomerular necrosis and peritubular inflammation; Δ*hisD*—minor inflammatory foci.

To evaluate the role of *hisD* in *S. aureus* pathogenicity, we compared the median lethal dose (LD_50_) of wild-type (WT) and Δ*hisD* strains in BALB/c mice via intraperitoneal challenge. The LD_50_ for Δ*hisD* (4.17 × 10^9^ CFU/mL) was 5.6-fold higher than that of WT (7.42 × 10^8^ CFU/mL). Mortality reached 100% at inocula ≥2.10 × 10^9^ CFU/mL for WT, whereas Δ*hisD* required ≥1.33 × 10^10^ CFU/mL to achieve full lethality. Notably, the Δ*hisD* strain exhibited no lethality at a dose of ≤3.33 × 10^9^ CFU/mL, in sharp contrast to the wild-type strain, which remained non-lethal even at 2.63 × 10^8^ CFU/mL (Fig. 4A). Consistent with reduced lethality, Δ*hisD*-infected mice exhibited significantly lower bacterial burdens in the liver, spleen, kidneys, and lungs compared with WT-infected mice (*P* < 0.05) 7 days post-inoculation (1.5 × 10^8^ CFU/mL, 600 µL) (Fig. 4B). Histopathological analysis revealed severe inflammation, suppurative necrosis, and tissue architecture disruption in WT-infected organs. In contrast, Δ*hisD* infection resulted in markedly attenuated inflammatory responses and minimal tissue damage (Fig. 4C). These findings conclusively demonstrate that *hisD* deletion cripples *S. aureus* virulence *in vivo*.

### Pixantrone demonstrates high-affinity binding to HisD through structural and energetic complementarity

**Fig 5:**
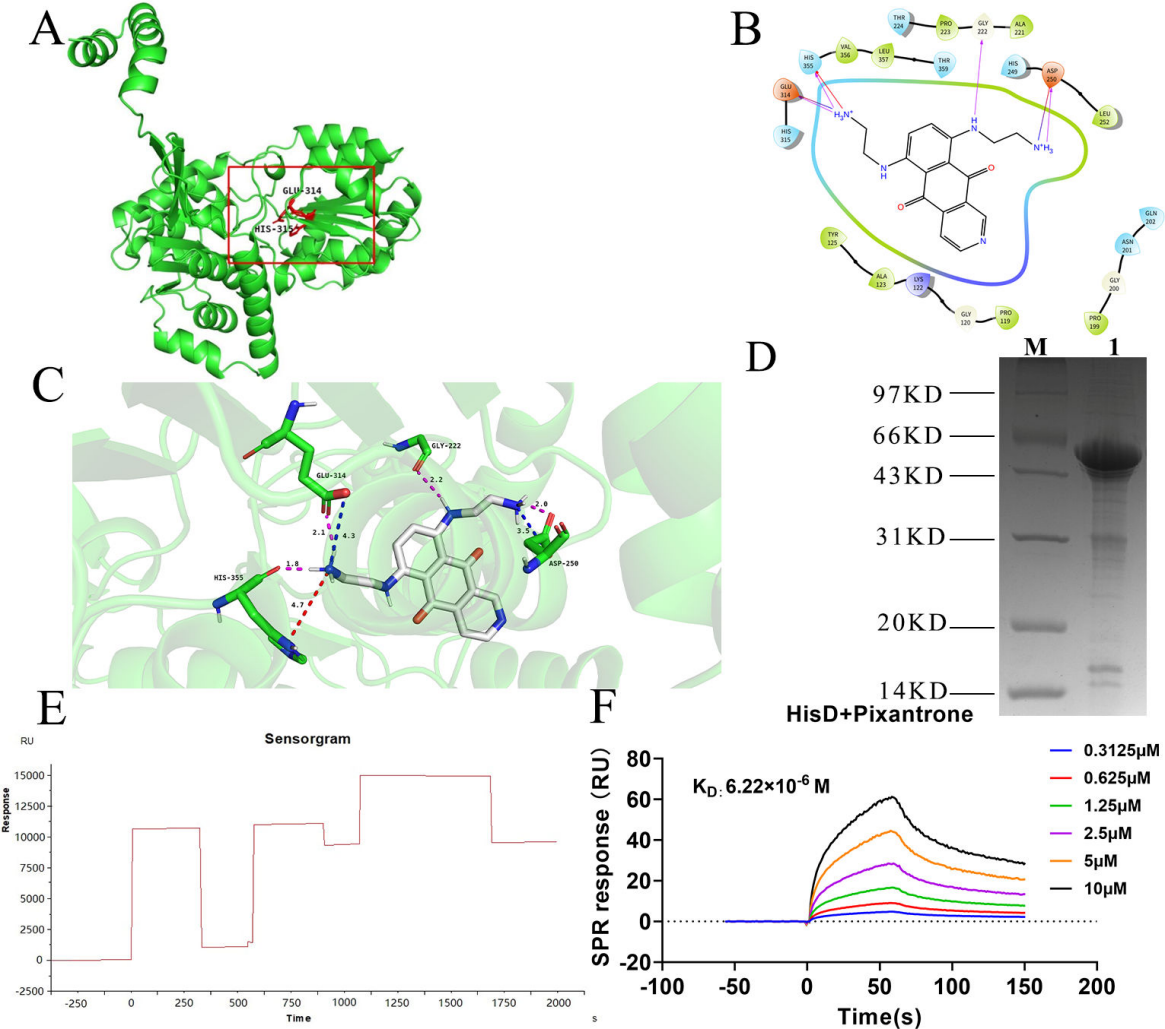
Virtual molecular docking of HisD with pixantrone and surface plasmon resonance (SPR) binding validation. (**A**) AlphaFold-predicted tertiary structure of HisD (UniProt ID: A0A0H3KAR9; residues 1–416), highlighting the active site (Glu314, Lys315) used for structure-based virtual screening. (**B**) Two-dimensional schematic of pixantrone-HisD interactions. (**C**) Three-dimensional binding model showing pixantrone (gray/white sticks) within the HisD active site (green cartoon). Key interactions: hydrogen bonds (magenta dashed lines, *n* = 4), salt bridges (blue dashed lines, *n* = 2), and a π-cation interaction (red dashed line). Atom colors: nitrogen (blue), oxygen (red), hydrogen (white). (**D**) 12% SDS-PAGE electrophoresis pattern of HisD protein purification, Molecular weight 53 KD, M: Marker, 1: Purified HisD protein. (**E**) HisD immobilization on the CM5 sensor chip (coupled response: 9423 RU). (**F**) SPR sensograms of HisD-pixantrone binding.

To identify HisD-targeted inhibitors, we conducted structure-based virtual screening using the Schrödinger Maestro 12.8 platform with the Glide docking module. From the HY-L001P Bioactive Compound Library Plus (https://www.medchemexpress.cn/screening/Bioactive_Compound_Library_Plus.html) and HY-L032 Fragment Library (https://www.medchemexpress.cn/screening/Fragment_Library.html), pixantrone—a nitrogenated anthraquinone derivative—emerged as a top candidate, exhibiting a Glide docking score of −10.106 kcal/mol (Fig. 5A), indicative of strong thermodynamic favorability. The predicted binding mode revealed pixantrone occupying the HisD active site (Glu314/Lys315) through four hydrogen bonds, two salt bridges, and one π-cation interaction (Fig. 5B and C). This multi-modal interaction network stabilizes pixantrone within a hydrophobic pocket formed by HisD’s β-sheet core, suggesting competitive inhibition of substrate access. The compound’s planar anthraquinone scaffold aligns with the enzyme’s catalytic groove, whereas its protonated amino groups engage in charge complementarity with acidic residues—a mechanism consistent with anthraquinone-derived inhibitors targeting NAD^+^-dependent enzymes. To validate the HisD-pixantrone binding affinity, we expressed and purified HisD (molecular weight: 53 KD) (Fig. 5D) and quantified interactions by surface plasmon resonance (SPR). Following confirmation of HisD immobilization on a CM5 sensor chip (Fig. 5E), SPR analysis yielded a dissociation constant K_D_ of 6.22 × 10^−6^ M for the HisD-pixantrone complex (Fig. 5F), confirming strong mutual affinity.

### Pixantrone dose-dependently suppresses virulence gene expression and hemolytic activity in *S. aureus* laboratory and clinical strains

**Fig 6:**
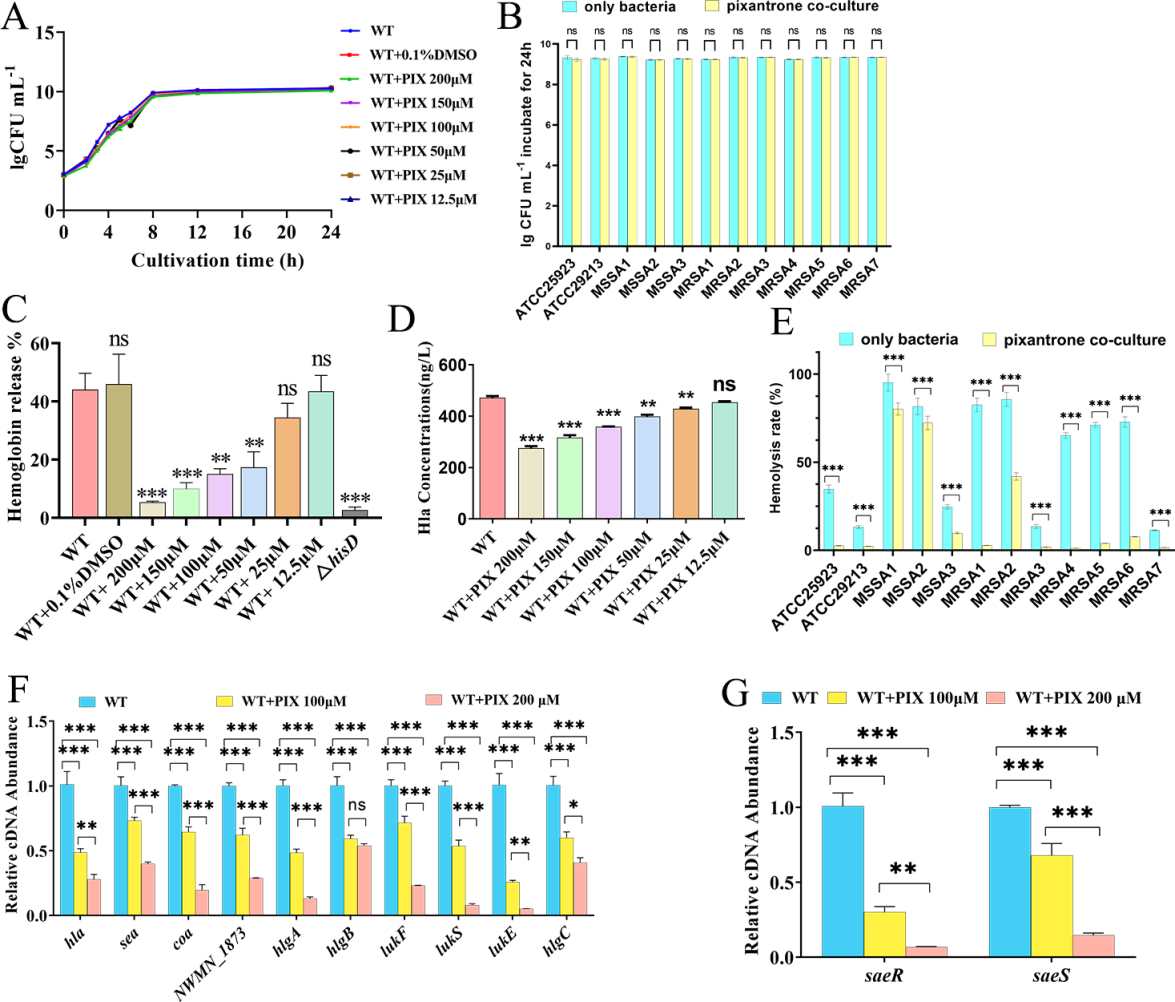
Dose-dependent inhibition of *S. aureus* virulence by pixantrone. (**A**) Growth kinetics of *S. aureus* Newman WT treated with pixantrone (0–200 µM). (**B**) 12 clinical strains cultured in TSB for 24h with or without pixantrone (200μM). (**C**) Hemolysis rates and (**D**) α-hemolysin (Hla) secretion (ELISA) in pixantrone-treated Newman cultures. (**E**) Hemolytic activity of clinical strains after 24 h incubation ±200 µM pixantrone. (**F and G**) qRT-PCR analysis of (**F**) virulence genes (*hla*, *coa*, *NWMN_1873*, *hlgABC*, and *lukEFS*) and (**G**) *saeR/S* expression. *P < 0.05, **P < 0.01, ***P < 0.001, ns *P* > 0.05.

We evaluated pixantrone’s anti-virulence efficacy against *S. aureus* Newman (laboratory strain) and clinical isolates using escalating concentrations (0–200 µM). In Newman, pixantrone exhibited no bactericidal activity (growth kinetics Fig. 6A) but significantly reduced hemolytic activity at ≥50 µM (*P* < 0.05) and α-hemolysin (Hla) secretion at ≥25 µM (*P* < 0.05) vs untreated controls (Fig. 6C and D). No inhibition occurred at <25 µM. For clinical strains (ATCC 25923, ATCC 29213, three MSSA, seven MRSA), 200 µM pixantrone in TSB for 24 h showed no growth inhibition (*P* > 0.05; Fig. 6B). However, hemolysis assays revealed significantly reduced hemolytic activity in all 12 strains versus untreated controls (*P* < 0.05; Fig. 6E). qRT-PCR demonstrated dose-dependent downregulation of virulence genes (*hla*, *coa*, *NWMN_1873*, *hlgABC*, *lukEFS*) and the *saeR/S* regulatory system at 100–200 µM (*P* < 0.05; Fig. 6F and G). Treatment with 200 µM pixantrone for 24 h in TSB co-culture significantly suppressed the expression of most virulence-related genes (*P* < 0.05). Exceptions included *lukE* (in MSSA 1, 2, and MRSA 1, 2, 3, 4, 5, 7), *saeR* (in MRSA 5), *saeS* (in MSSA 1 and MRSA 5), *NWMN_1873* (in MSSA 1 and 2μ), and *hlgA* (in MSSA 2), which were not significantly affected, as detailed in Fig. S1. Collectively, pixantrone suppresses *S. aureus* virulence via transcriptional regulation without affecting growth.

### Pixantrone dose-dependently suppresses biofilm formation in the *S. aureus* Newman strain

**Fig 7:**
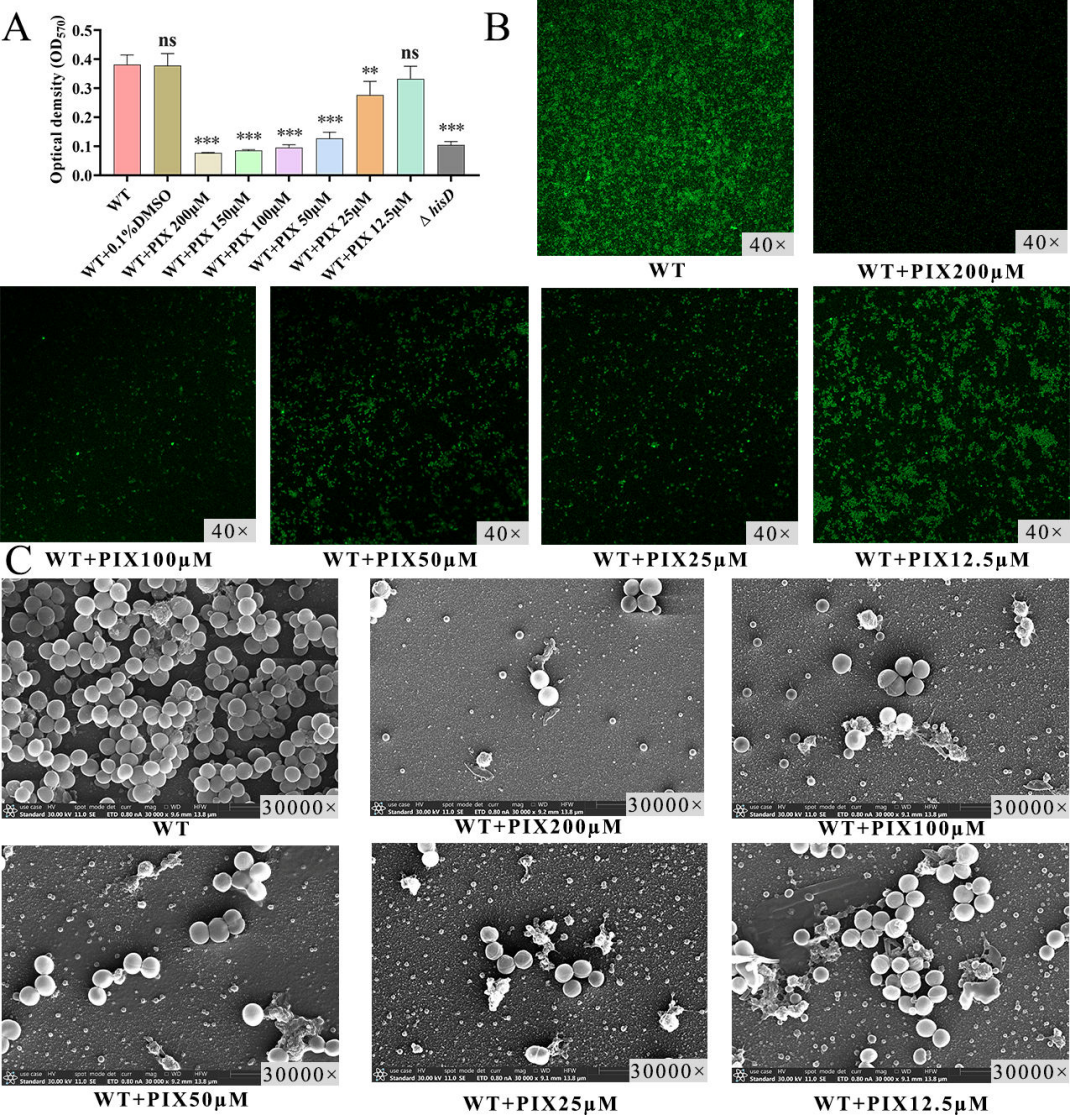
Dose-dependent suppression of *S. aureus* Newman strain biofilm formation by pixantrone. (**A**) Biofilm biomass quantified by crystal violet staining (OD_570nm_). Compared with WT, **P < 0.01, ***P < 0.001, ns *P* > 0.05. (**B**) LSCM of FITC-CoA-stained biofilms (green). (**C**) SEM of biofilms. Untreated WT formed dense biofilms with extracellular matrix, whereas pixantrone dose-dependently reduced cellular aggregation and matrix deposition.

We conducted a quantitative analysis and microscopic observation of the biofilm formation of the *S. aureus* Newman strain after 24 h incubation with pixantrone (0–200 µM) using crystal violet staining, LSCM, and SEM. Biofilm biomass (OD_570nm_) decreased at 25–200 µM (*P* < 0.05), with maximal inhibition at 200 µM (Fig. 7A). Biofilm biomass (OD_570nm_) decreased significantly at concentrations ≥ 25 µM (*P* < 0.05), with maximal inhibition at 200 µM (Fig. 7A). LSCM and SEM analyses confirmed progressive degradation of biofilm architecture with increasing drug concentrations, characterized by disrupted structural integrity, reduced cellular density, and diminished extracellular matrix deposition (Fig. 7B and C).

### Pixantrone attenuates systemic inflammation and skin pathogenesis in *S. aureus*-infected BALB/c mice

**Fig 8:**
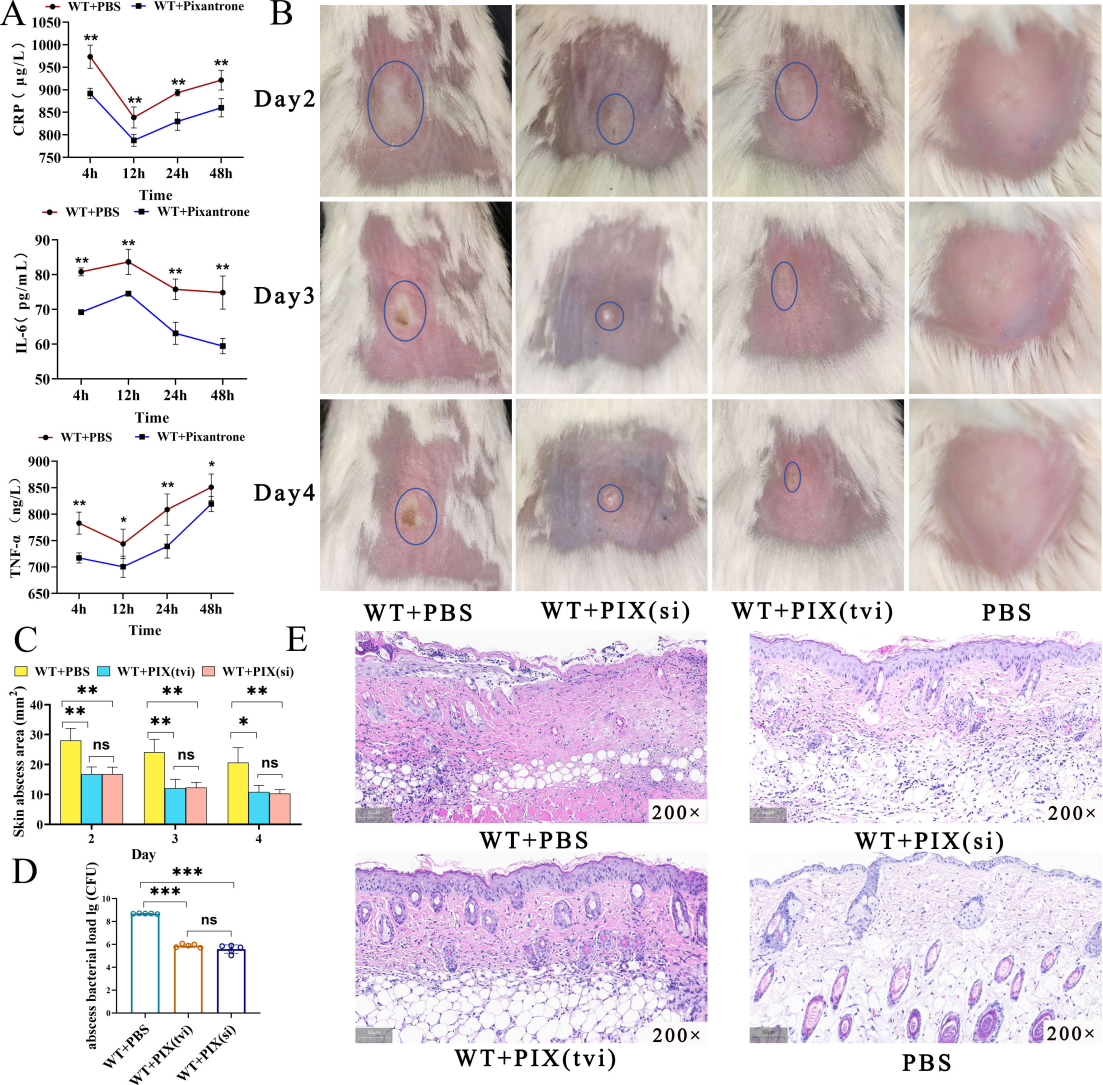
Therapeutic efficacy of pixantrone against *S. aureus* skin or intraperitoneal infection in BALB/c mice. (**A**) Serum levels of proinflammatory cytokines (IL-6, TNF-α) and acute-phase proteins (CRP) were quantified via ELISA at 4, 12, 24, and 48 h post-intraperitoneal infection with *S. aureus* (1.5 × 10^8^ CFU/mL, 500 µL per mouse). Treatment groups: PBS (control), pixantrone (30 mg/kg, single dose administered). Data represent mean ± SD (*n* = 12 per group). *P < 0.05, **P < 0.01. (**B**) Representative skin abscesses at day 4 post-infection. WT + PBS developed persistent lesions (blue circle), whereas WT + PIX(si) and WT + PIX(tvi) exhibited reduced lesion size (blue circle) (*n* = 5 per group). (**C**) Quantification of abscess area over 3 days. WT + PIX groups showed a reduction in lesion size vs. WT + PBS (*P < 0.05, **P < 0.01, ns *P >* 0.05). (**D**) The bacterial load in skin abscess tissue on the fourth day of infection was significantly lower in the WT + PIX(si) and WT + PIX(tvi) groups than in the WT + PBS group (***P < 0.001, ns *P >* 0.05). (**E**) H&E-stained skin sections: WT + PBS—disrupted—disrupted epidermal architecture, neutrophilic infiltration, and dermal necrosis; WT + PIX(si) and WT + PIX(tvi) groups—partial re-epithelialization, organized dermal layers, and residual inflammation (*n* = 3 per group).

To assess the anti-inflammatory efficacy of pixantrone *in vivo*, BALB/c mice were intraperitoneally challenged with a sublethal dose of wild-type *S. aureus* (1.5 × 10^8^ CFU/mL, 500 μL per mouse), followed by intravenous administration of pixantrone (30 mg/kg) at 1 h post-infection. Serum analysis revealed that pixantrone significantly suppressed proinflammatory mediators across all time points (4, 12, 24, 48h), reducing C-reactive protein (CRP), interleukin-6 (IL-6), and tumor necrosis factor-α (TNF-α) compared with PBS-treated controls (*P* < 0.05) (Fig. 8A). We next evaluated pixantrone’s therapeutic potential in a subcutaneous *S. aureus* infection model. Mice receiving concurrent subcutaneous (WT + PIX(si)) or intravenous (WT + PIX(tvi)) pixantrone (30 mg/kg) exhibited smaller abscess areas on days 2–4 post-infection versus untreated controls (WT + PBS; *P* < 0.05), with no significant difference between administration routes (*P* > 0.05) (Fig. 8B and C). Bacterial burdens in abscess homogenates were lower in both pixantrone groups versus WT + PBS (***P < 0.001) (Fig. 8D). Histopathological analysis of day 4 lesions demonstrated that pixantrone treatment mitigated tissue damage: WT + PBS: disrupted epidermal architecture, neutrophilic infiltration, and dermal necrosis. WT + PIX (si/tvi): partial re-epithelialization, organized keratinocyte layers, and residual peri-vascular inflammation (Fig. 8E).

## DISCUSSION

*S. aureus* remains a formidable pathogen in both healthcare and community settings, with increasing antibiotic resistance underscoring the urgent need for innovative anti-infective strategies (21). Our study identifies histidinol dehydrogenase (HisD)—a Zn^2+^-dependent enzyme catalyzing the terminal steps of histidine biosynthesis—as a critical regulator of *S. aureus* virulence. The Δ*hisD* strain exhibited no growth defect on nutrient-rich TSA/TSB medium. However, following cultivation in this medium, it demonstrated a significant reduction in hemolytic activity, secretion of α-hemolysin, and biofilm formation. Concurrently, the strain downregulated key virulence-associated genes (*hla*, *coa*, *hlgABC*, and *lukEFS*), as well as the *saeR/S* two-component regulatory system. In addition, expression of the genes encoding Aur and Scap—proteins known to inhibit biofilm formation (22)—was upregulated in the Δ*hisD* mutant. Although the Δ*hisD* strain failed to grow in the basal medium, growth was restored upon supplementation with histidine. Notably, the Δ*hisD* mutant exhibited a 5.6-fold increase in murine LD_50_ and reduced organ bacterial burdens, demonstrating *hisD*’s central role in *S. aureus* pathogenicity.

Although histidine biosynthesis is essential across bacteria, its linkage to virulence remains underexplored. Our findings align with prior reports in *Brucella* and *Mycobacterium tuberculosis* where HisD functions as both a virulence factor and therapeutic target (15–17, 23). Intriguingly, histidine auxotrophy in *Pseudomonas aeruginosa* similarly restricts growth and virulence (24), suggesting the evolutionary conservation of histidine metabolism in bacterial pathogenesis.

The attenuated virulence of Δ*hisD* likely stems from dual effects: toxin dysregulation and biofilm impairment. Reduced expression of cytotoxins (*hla*, *hlgABC*), leukocidins (*lukEFS*), and coagulase (*coa*)—hallmarks of invasive *S. aureus* strains (25–29)—implies histidine scarcity affects the downregulation of toxin transcription levels. Histidine biosynthesis intersects with nitrogen/carbon metabolism and glutamate synthesis—processes essential for biofilm matrix production (30–32). Combining azithromycin with *xylose-fermenting Staphylococcus* can reduce the expression of proteins necessary for histidine biosynthesis, which in turn impacts biofilm formation (33). Additionally, a reduction in histidine synthesis can indirectly influence glutamate synthesis, which has been confirmed to affect bacterial biofilm formation in various species, such as *S. aureus* and *Bacillus subtilis* (34, 35).

To investigate potential links between *hisD* downregulation and attenuated virulence, we quantified mRNA levels of 16 TCS-encoding genes in *S. aureus* Newman wild-type (WT) and Δ*hisD* strains. Among *S. aureus* TCS regulators, Agr (36), SaeRS (37), and ArlSR (38) constitute core virulence determinants. WalKR (39) and VraSR (40, 41) mediate antibiotic resistance, whereas SrrAB (42), NreBC, and HssRS (43, 44) enable survival in hypoxic host niches. LytSR senses membrane damage to modulate autolysin expression—directing cell lysis and biofilm dispersion (45). Quantification revealed no differential expression of *agrA/agrC* (Agr), *NWMN_1327*/*NWMN_1328* (Arl), *lytS/lytR* (LytSR), *NWMN_0017*/*NWMN_0018* (WalkR), *srrA/srrB* (SrrAB), *NWMN_0939* (AirSR), *NWMN_2291* (NreBC), or *NWMN_2263*/*NWMN_2264* (HssRS) between strains. Notably, *saeR/saeS* (Sae) expression was significantly reduced in the Δ*hisD* strains versus WT (*P* < 0.05), and this attenuation was rescued upon genetic complementation of *hisD*. The SaeRS two-component system serves as a master controller of *S. aureus* pathogenicity, coordinating the expression of over 20 virulence determinants, including pore-forming toxins (α-hemolysin, leukocidins), immune evasion molecules (protein A), and tissue-degrading proteases, especially in antibiotic-resistant strains (46). The common presence of *saeR* and *saeS* genes in MRSA and vancomycin-resistant *S. aureus* (VRSA) points to a correlation between these genes and the virulence as well as the pathogenesis of *S. aureus* (46). Our discovery that *hisD* deletion downregulates *saeR/S* expression unveils an unexpected metabolic-virulence nexus. We propose one mechanistic hypothesis: Histidine biosynthesis may supply the phosphorylatable histidine residue essential for SaeS (sensor kinase) autophosphorylation. Depleted histidine pools in Δ*hisD* could impair SaeS→SaeR phosphotransfer, attenuating virulence gene activation (47, 48). HisD-generated α-ketoglutarate—a key TCA cycle intermediate—might modulate *saeR/S* expression via carbon catabolite repression (CcpA) or CodY regulons (49, 50). This study observed a significant decrease in the expression of *saeR* and *saeS* following the knockout of the *hisD* gene, indicating a relationship between *hisD* and both *saeR/S*, which subsequently influences the virulence of *S. aureus*.

Through structure-based virtual screening, we identified PIX, a nitrogenated anthraquinone derivative, as a potent HisD inhibitor. Molecular docking revealed PIX occupies the HisD catalytic pocket through four hydrogen bonds, two salt bridges, and one π-cation interaction with key residues (Glu314, His355, Gly222, and Asp250). Surface plasmon resonance (SPR) validation confirmed strong HisD-PIX binding, yielding a dissociation constant K_D_ of 6.22 × 10^−6^ M. *In vitro*, PIX dose-dependently suppressed hemolysis, biofilm formation, and virulence gene expression without affecting bacterial growth, confirming its anti-virulence specificity. In murine models, PIX (30 mg/kg i.v.) reduced inflammatory markers (CRP, IL-6, TNF-α), abscess size, and bacterial loads to levels comparable with Δ*hisD* infection. Notably, pixantrone is a clinically approved anthraquinone derivative used in monotherapy for aggressive B-cell non-Hodgkin lymphoma (European Medicines Agency [EMA]-approved) (51, 52). Its structural modification—lacking the quinone-hydroquinone iron-binding site—reduces anthracycline-associated cardiotoxicity. Nitrogen substitution enhances DNA affinity through strengthened hydrogen bonding and basicity (51–53). Although PIX’s antitumor mechanisms are well characterized, its antibacterial applications remain unexplored. This dosage of 30 mg/kg intravenous infusion—adapted from PIX’s oncology regimen (54–56)—achieved efficacy without overt toxicity, leveraging its unique DNA intercalation chemistry (absence of quinone redox cycling) (57, 58). It is expected to serve as an adjunctive treatment for severe *S. aureus* infections. This presents a novel strategy for treating *S. aureus* infections. However, this study has not yet examined how histidine synthesis is linked to *Staphylococcus* virulence. Moreover, additional research is necessary to establish the best dosage and administration method for PIX and its potential for combination therapy with other antibiotics.

In conclusion, histidinol dehydrogenase (HisD), a key enzyme in histidine synthesis, plays an essential role in regulating *S. aureus* virulence and may serve as a new target for developing anti-virulence therapies. Pixantrone, as an inhibitor of HisD, is a potential drug for combating the virulence of *S. aureus*.

## MATERIALS AND METHODS

### Bacterial strains cultivation and genetic complementation

All bacterial strains and plasmids used in this study are listed in Table S1. All *S. aureus* strains were cultured in Tryptic Soy Agar (TSA) or Broth (TSB) (Solarbio, Beijing, China) under static or shaking conditions (180 rpm), respectively, at 37°C for 16–18 h. *E. coli* strains DC10B and DH5α were propagated in Lysogeny Broth (LB; Solarbio) with 180 rpm agitation at 37°C. The *tet*-inducible shuttle plasmid pRAB11 (harboring the anhydrotetracycline (Atc)-regulated *tet* operon) was employed for genetic complementation. For optimal *hisD* expression, strains Δ*hisD*::pRAB-*hisD* (complemented), WT::pRAB-*hisD* (overexpression), and empty vector controls (Δ*hisD*::pRAB11, WT::pRAB11) were cultured in TSB supplemented with 100 ng/mL Atc for 24 h at 37°C with aeration (59). To confirm the Δ*hisD* strain is histidine-deficient, 20 µL of Δ*hisD* bacterial suspension (37°C, 200 rpm, 24 h) was evenly spread onto two types of agar plates: a minimal medium and a minimal medium supplemented with histidine (60).

### Gene knockout via homologous recombination

The *hisD* deletion mutant (Δ*hisD*) was constructed using the temperature-sensitive plasmid pBT2 through homologous recombination (61, 62). Primers *hisD*-uf/ur and *hisD*-df/dr (Table S2), designed via SnapGene based on *hisD* flanking sequences and pBT2 backbone, amplified ~1 kb upstream/downstream regions of *hisD* from *S. aureus* Newman genomic DNA using Q5 High-Fidelity DNA Polymerase (Takara Bio, Japan). The pBT2 plasmid, extracted from *E. coli* DH5α, was digested with BamHI/SalI (Thermo Fisher Scientific, USA) and ligated to PCR fragments using ClonExpress-II (Vazyme, China). The recombinant plasmid pBT2-Δ*hisD* was electroporated into *S. aureus* RN4220 (Bio-Rad GenePulser: 2.9 kV, 25 µF, 100 Ω) for methylation, followed by transfer into the Newman strain. Allelic replacement was achieved through temperature shift: plasmid integration at 42°C and excision at 25°C. Successful *hisD* deletion was confirmed by DNA sequencing (BGI Genomics, China).

### Complementation and overexpression strains construction

The *tet*-inducible plasmid pRAB11 was employed to generate complemented (Δ*hisD*::pRAB-*hisD*) and overexpression (WT::pRAB-*hisD*) strains. The *hisD* ORF was amplified with primers *hisD*-f/r (Table S2), cloned into SalI/KpnI-digested pRAB11, and transformed into *E. coli* DC10B. Recombinant plasmid pRAB-*hisD* was electroporated into *S. aureus* Newman and Δ*hisD* strains as above. Empty vector controls (WT::pRAB11, Δ*hisD*::pRAB11) were generated in parallel. All strains were cultured in TSB supplemented with 100 ng/mL anhydrotetracycline (Atc; Solarbio) for 24 h to induce *hisD* expression, validated by DNA sequencing (BGI Genomics, China).

### Bacterial growth curves

Overnight cultures of *S. aureus* Newman WT or Δ*hisD* were diluted to 1 × 10^3^ CFU/mL in fresh TSB. Initial CFU counts were validated via serial dilution plating. Cultures were incubated aerobically at 37°C with orbital shaking (180 rpm), and viable counts were determined every 2 h over 24 h using standardized drop-plate methodology (63).

### Bacterial growth assessment of clinical *S. aureus* strains following pixantrone exposure

Clinical *S. aureus* strains (ATCC 25923, ATCC 29213, three MSSA isolates, seven MRSA isolates) were incubated with or without 200 µM pixantrone in TSB for 24 h at 37°C. Bacterial viability was quantified by standard plate counts to determine pixantrone’s effect on growth kinetics.

### MIC determination

MIC values for ampicillin (β-lactam), levofloxacin (fluoroquinolone), and vancomycin (glycopeptide) (Solarbio, Beijing, China) were assessed via broth microdilution following Clinical and Laboratory Standards Institute (CLSI) guidelines (64, 65). Briefly, incubate the 1:10^3^ diluted bacterial culture at 37°C with shaking at 180 rpm for 3 h, and then further dilute it to a ratio of 1:10 to prepare the working bacterial solution. Serial 2-fold antibiotic dilutions (0.016–256 µg/mL) were prepared in sterile 96-well plates, inoculated with 100 µL bacterial suspension, and incubated statically at 37°C for 24 h. The MIC was defined as the lowest concentration demonstrating complete visual inhibition of bacterial growth.

### Hemolytic activity assessment on blood agar

Bacterial strains were streaked onto sheep blood agar (SBA; 5% defibrinated sheep blood, Solarbio, China) plates and incubated aerobically at 37°C. Hemolytic zones surrounding colonies were observed at 12 h post-inoculation, with continued observation at 12 h intervals for 36 h (66).

### Quantitative hemolysis assay

*S. aureus* suspensions (24 h cultures) were centrifuged at 10,000 × *g* for 2 min at 4°C. Supernatants were diluted 1:4 in sterile PBS and mixed with 2.5% (vol/vol) defibrinated sheep erythrocytes (25 µL) in 96-well plates. Controls included Triton X-100 (100% hemolysis; positive) and PBS (0% hemolysis; negative). Following 1 h incubation at 37°C, samples were centrifuged at 6,000 × *g* for 5 min to pellet intact erythrocytes. Hemoglobin release was quantified by measuring supernatant absorbance at 570 nm. Hemolysis percentage was calculated as: hemolysis % = [(*A*570 sample − *A*570 negative)/(*A*570 positive − *A*570 negative)] × 100% (67). Clinical strains were incubated in TSB ± 200 µM pixantrone for 24 h at 37°C, followed by supernatant collection for hemolytic activity quantification.

### ELISA quantification of α-hemolysin (Hla) and inflammatory markers (IL-6, TNF-α, and CRP)

Levels of *S. aureus* α-hemolysin (Hla; Qiming Biotech, Shanghai, China) and host inflammatory markers (IL-6, TNF-α, and CRP; Meimian Biotechnology, Jiangsu, China) were quantified using commercial enzyme-linked immunosorbent assay (ELISA) kits according to manufacturer protocols. Briefly, 100 µL of cell culture supernatant or murine serum samples were added to antibody-precoated 96-well plates and incubated for 0.5 h at 37°C. Following plate washing, horseradish peroxidase (HRP)-conjugated detection antibodies were applied for 0.5 h at 37°C. Tetramethylbenzidine (TMB) substrate was added, and reactions were stopped with the stop buffer. Absorbance at 450 nm (OD_450nm_) was measured using an MB16-414 microplate reader (Jingfu Instruments Co., Ltd., Shanghai, China). Standard curves were generated using serially diluted recombinant proteins, with four-parameter logistic regression applied for concentration interpolation. All samples were analyzed in triplicate.

### Biofilm biomass quantification

Biofilm formation was assayed in 96-well polystyrene plates as previously described (67) with modifications. Briefly, 2 µL of overnight *S. aureus* cultures (OD_600nm_ = 1.0) were inoculated into 200 µL TSB supplemented with 0.5% (wt/vol) glucose. For strains harboring pRAB11-derived plasmids, 0.1 µg/mL Atc was added to induce gene expression. Plates were incubated statically at 37°C for 24 h. After PBS washing, biofilms were fixed with 100 µL methanol (15 min), stained with 1% (wt/vol) crystal violet (15 min), and solubilized with 33% (vol/vol) glacial acetic acid. Biomass was quantified by measuring absorbance at 570 nm (OD_570nm_) using an MB16-414 microplate reader (Jingfu Instruments, China).

### Biofilm morphological analysis using SEM

Biofilms grown on glass coverslips were fixed with 2.5% (vol/vol) glutaraldehyde in 0.1 M PBS (pH 7.4) at 4°C overnight. Samples were sequentially dehydrated in ethanol gradients (30%, 50%, 70%, 90%, and 100%), critical-point dried, and sputter-coated with 10 nm gold-palladium. Imaging was performed using a Thermo Scientific Apreo S SEM (Thermo Fisher Scientific, USA) at 5 kV.

### Biofilm morphological analysis using LSCM

Biofilms were stained with 50 µg/mL FITC-conjugated concanavalin A (FITC-ConA; Sigma-Aldrich) in PBS (4°C, 30 min, dark) to visualize polysaccharide-rich matrix components. After PBS washing, biofilms were imaged using a Zeiss LSM 900 confocal microscope (Carl Zeiss AG, Germany) with a 488 nm excitation laser and 500−550 nm emission filter. Z-stack images were processed via ZEN 3.4 software.

### RNA extraction and quantitative reverse-transcription PCR (qRT-PCR)

*S. aureus* strains were cultured in TSB at 37°C with 180 rpm shaking. For strains harboring pRAB11-derived plasmids, 0.1 µg/mL Atc was supplemented to induce gene expression. After 24 h, bacterial pellets were collected by centrifugation at 12,000 × *g* for 2 min (4°C), treated with lysostaphin (32 U/mL) at 37°C for 1 h, and total RNA was extracted using the FastPure Cell/Tissue Total RNA Isolation Kit (Accurate Biology, Hunan, China). RNA purity and concentration were verified via NanoDrop OneC spectrophotometry (Thermo Fisher Scientific). RNA (1 µg) was reverse-transcribed into cDNA using TransScript One-Step gDNA Removal and cDNA Synthesis SuperMix (TransGen Biotech, Beijing, China). qRT-PCR was performed on a QuantStudio 5 Real-Time PCR System (Thermo Fisher Scientific) with the following 20 µL reaction mixture: 2 µL gene-specific primers (10 µM), 2 µL cDNA template (20 ng/µL), and 10 µL 2 × TransStart Tip Green qPCR SuperMix (TransGen Biotech). Thermal cycling conditions: 95°C for 2 min (initial denaturation); 40 cycles of 95°C for 5 s, 60°C for 15 s, and 72°C for 30 s; followed by melt curve analysis (60°C–94°C, 0.3 °C/s increment). *16S rRNA* served as the endogenous control (Ct < 37). Relative gene expression was calculated using the 2^-△△CT^ method. Statistical significance (*P* < 0.05) was determined via a two-tailed Student’s *t*-test, with data visualized as box plots using GraphPad Prism 9.0. All primers used in this study are listed in Table S3.

### Structure-based virtual screening of HisD inhibitors

The predicted three-dimensional structure of *S. aureus* HisD (UniProt ID: A0A0H3KAR9) was retrieved from the AlphaFold Protein Structure Database (version 2.3.2). Hydrogen atoms were added, and the structure was energy-minimized using the Protein Preparation Wizard module in Schrödinger Suite 2022-3 with the OPLS_2005 force field. Structural optimization included bond order assignment, protonation state adjustment at pH 7.4, and removal of steric clashes (target RMSD < 0.30 Å). A receptor grid (20 × 20 × 20 Å³) was centered on the catalytic residues Glu314 and Lys315 using the Glide Receptor Grid Generation module. Two commercial compound libraries (HY-L001P Bioactive Compound Library Plus and HY-L032 Fragment Library; MedChemExpress, China) were preprocessed via the LigPrep workflow: ionization states were generated at pH 7.0 ± 2.0 using Epik, stereoisomers enumerated, and low-energy conformers minimized with OPLS_2005. Compounds were docked against the HisD active site using the Glide module (Schrödinger) in three sequential modes: high-throughput virtual screening (HTVS), standard precision (SP), and standard precision (SP). Top-ranked compounds were selected based on docking scores (kcal/mol) and binding pose analysis.

### HisD protein’s expression and purification

The recombinant plasmid pET30a-*hisD* was transformed into *E. coli* BL21 competent cells via heat shock (42°C, 45 s). Transformed cells were cultured in LB with 50 µg/mL kanamycin at 37°C with shaking (220 rpm) until OD_600_ reached 0.6–0.8. Protein expression was induced with 0.5 mM IPTG at 16°C for 16 h. Cells were lysed by sonication in 50 mM NaH_2_PO_4_, 300 mM NaCl, 10 mM imidazole (pH 8.0). HisD from inclusion bodies was solubilized in 8 M urea buffer and purified by Ni-NTA chromatography under denaturing conditions, followed by stepwise urea dialysis. Refolded protein was concentrated and buffer-exchanged into PBS, yielding 85% pure HisD (confirmed by SDS-PAGE) at 0.5 mg/mL (68).

### Surface plasmon resonance (SPR) analysis

Biomolecular interactions were analyzed using a Biacore 8K instrument (Cytiva). HisD protein was immobilized on a CM5 sensor chip via amine coupling. Before immobilization, channel 2 of the chip was activated at a flow rate of 10 µL/min with 1-ethyl-3-(3-dimethylaminopropyl) carbodiimide (EDC, GE Healthcare) and N-hydroxysuccinimide (NHS, GE Healthcare). HisD was diluted to 50 µg/mL in 10 mM sodium acetate and immobilized on flow cell 2 at 10 µL/min for 7 min. Pixantrone (0.78–50 µM) was injected at 30 µL/min for 150 s in ascending concentration order. Surface regeneration was performed after each analyte injection using 10 mM glycine-HCl (pH 2.0) with a 60 s contact time. Sensorgrams were globally fitted to a 1:1 Langmuir binding model in Biacore Insight Evaluation Software (Cytiva, Marlborough, MA, USA) to determine equilibrium dissociation constants K_D_ (69).

### BALB/c mice

Female BALB/c mice (8 weeks old, 20–22 g) were obtained from the Laboratory Animal Center of Lanzhou Veterinary Research Institute, Chinese Academy of Agricultural Sciences, and housed under specific pathogen-free conditions (22°C ± 1°C, 55% humidity, 12 h light/dark cycle).

### Median lethal dose (LD_50_) determination

Mice were randomized into three groups: control (*n* = 5), intraperitoneal (i.p.) injection of sterile PBS. WT (*n* = 35); i.p. challenge with 0.6 mL *S. aureus* Newman WT (7-fold serial dilution in PBS; 2.63 × 10^8^–1.67 × 10^10^ CFU/mL); and Δ*hisD* (*n* = 35), i.p. challenge with Δ*hisD* strain (prepared as above; 2.09 × 10^8^–1.33 × 10^10^ CFU/mL). Mortality was monitored for 7 days. LD_50_ values were calculated via Probit analysis using SPSS 22.0.

### Bacterial organ burden and histopathology

Twenty-four mice were randomized into: control (*n* = 8): i.p. PBS; WT (*n* = 8): i.p. 0.6 mL WT (~1.5 × 10^8^ CFU/mL); and Δ*hisD* (*n* = 8), i.p. 0.6 mL Δ*hisD* (~1.5 × 10^8^ CFU/mL). At day 7 post-infection, mice were euthanized by CO_2_ asphyxiation. Liver, kidneys, spleen, and lungs were aseptically harvested, homogenized in PBS, and plated on TSA for CFU enumeration (five in each group). Mice tissues from each group were fixed in 4% paraformaldehyde, paraffin-embedded, sectioned, and stained with hematoxylin and eosin (H&E) for histopathological evaluation (*n* = 3).

### Cutaneous abscess model

Thirty-two mice were randomized into four groups (*n* = 8/group): (i) WT + PIX (tvi), subcutaneous (s.c.) WT (1.5 × 10^8^ CFU/mL in 100 µL PBS) + single i.v. pixantrone (30 mg/kg) at 1 h post-infection. (ii) WT + PIX (si), s.c. WT + daily s.c. pixantrone (10 mg/kg) at the infection site (days 1–3). (iii) WT + PBS: s.c. WT + PBS. (iv) PBS control: s.c. PBS. Abscess dimensions (length *L*, width *W*) were measured daily with digital calipers, and area calculated as *A* = π *(L × W)*/2 (67). On day 4, abscesses were excised for CFU quantification or fixed for H&E staining (*n* = 3).

### Serum inflammatory marker profiling

Twenty-four mice were randomized into: (i) WT + PBS (*n* = 12), i.p. WT (1 × 10^8^ CFU/mL in 500 µL) + i .v. PBS. (ii) WT + PIX (*n* = 12), i.p. WT (1 × 10^8^ CFU/mL in 500 µL) + i .v. pixantrone (30 mg/kg). Retro-orbital blood was collected at 4, 12, 24, and 48 h post-treatment. Serum levels of IL-6, TNF-α, and CRP were quantified by ELISA.

### Statistical analysis

Experimental datas were analyzed using IBM SPSS Statistics 22.0 and GraphPad Prism 9.5.1. Continuous variables are expressed as mean ± standard deviation (SD). Between-group comparisons were performed using a two-tailed unpaired Student’s *t*-test for normally distributed data, with *P* < 0.05 considered statistically significant. For multiple comparisons, one-way ANOVA followed by Tukey’s post hoc test was applied where appropriate.

## SUPPLEMENTAL MATERIAL

Supplemental material spectrum.01429-25-s0001.docx Fig. S1; Tables S1 to S3.

ASM does not own the copyrights to Supplemental Material that may be linked to, or accessed through, an article. The authors have granted ASM a non-exclusive, world-wide license to publish the Supplemental Material files. Please contact the corresponding author directly for reuse.

## References

[1] Tong SYC, Fowler VG, Skalla L, Holland TL. 2025. Management of Staphylococcus aureus bacteremia: a review. JAMA 334:798–808. doi:10.1001/jama.2025.4288 40193249 PMC12663922

[2] Adedeji-Olulana AF, Wacnik K, Lafage L, Pasquina-Lemonche L, Tinajero-Trejo M, Sutton JAF, Bilyk B, Irving SE, Portman Ross CJ, Meacock OJ, Randerson SA, Beattie E, Owen DS, Florence J, Durham WM, Hornby DP, Corrigan RM, Green J, Hobbs JK, Foster SJ. 2024. Two codependent routes lead to high-level MRSA. Science 386:573–580. doi:10.1126/science.adn1369 39480932 PMC7617827

[3] Xue M, Chakraborty S, Gao R, Wang S, Gu M, Shen N, Wei L, Cao C, Sun X, Cai J. 2024. Antimicrobial guanidinylate polycarbonates show oral in vivo efficacy against Clostridioides difficile. Adv Healthc Mater 13:e2303295. doi:10.1002/adhm.202303295 38321619 PMC11144102

[4] Rasko DA, Sperandio V. 2010. Anti-virulence strategies to combat bacteria-mediated disease. Nat Rev Drug Discov 9:117–128. doi:10.1038/nrd3013 20081869

[5] Zhou S, Liu B, Zheng D, Chen L, Yang J. 2025. VFDB 2025: an integrated resource for exploring anti-virulence compounds. Nucleic Acids Res 53:D871–D877. doi:10.1093/nar/gkae968 39470738 PMC11701737

[6] Silva-Santana G. 2025. Staphylococcus aureus: dynamics of pathogenicity and antimicrobial-resistance in hospital and community environments - Comprehensive overview. Res Microbiol 176:104267. doi:10.1016/j.resmic.2025.104267 39805330

[7] Tam K, Torres VJ. 2019. Staphylococcus aureus secreted toxins and extracellular enzymes. Microbiol Spectr 7. doi:10.1128/microbiolspec.gpp3-0039-2018 PMC6422052 30873936

[8] Okuda K-I, Yamada-Ueno S, Yoshii Y, Nagano T, Okabe T, Kojima H, Mizunoe Y, Kinjo Y. 2022. Small-molecule-induced activation of cellular respiration inhibits biofilm formation and triggers metabolic remodeling in Staphylococcus aureus. mBio 13:e0084522. doi:10.1128/mbio.00845-22 35852317 PMC9426486

[9] Francis D, Bhairaddy A, Joy A, Hari GV, Francis A. 2023. Secretory proteins in the orchestration of microbial virulence: the curious case of Staphylococcus aureus. Adv Protein Chem Struct Biol 133:271–350. doi:10.1016/bs.apcsb.2022.10.004 36707204

[10] Witek W, Sliwiak J, Ruszkowski M. 2021. Structural and mechanistic insights into the bifunctional HISN2 enzyme catalyzing the second and third steps of histidine biosynthesis in plants. Sci Rep 11:9647. doi:10.1038/s41598-021-88920-2 33958623 PMC8102479

[11] Barbosa JARG, Sivaraman J, Li Y, Larocque R, Matte A, Schrag JD, Cygler M. 2002. Mechanism of action and NAD^+^-binding mode revealed by the crystal structure of l-histidinol dehydrogenase. Proc Natl Acad Sci USA 99:1859–1864. doi:10.1073/pnas.022476199 11842181 PMC122284

[12] Monti SM, De Simone G, D’Ambrosio K. 2016. l-histidinol dehydrogenase as a new target for old diseases. Curr Top Med Chem 16:2369–2378. doi:10.2174/1568026616666160413140000 27072690

[13] Grubmeyer C, Skiadopoulos M, Senior AE. 1989. l-histidinol dehydrogenase, a Zn^2+^-metalloenzyme. Arch Biochem Biophys 272:311–317. doi:10.1016/0003-9861(89)90224-5 2665648

[14] Abdo M-R, Joseph P, Boigegrain R-A, Montero J-L, Köhler S, Winum J-Y. 2008. Brucella suis histidinol dehydrogenase: synthesis and inhibition studies of substituted N-L-histidinylphenylsulfonyl hydrazide. J Enzyme Inhib Med Chem 23:357–361. doi:10.1080/14756360701617107 18569340

[15] Parish T. 2003. Starvation survival response of Mycobacterium tuberculosis. J Bacteriol 185:6702–6706. doi:10.1128/JB.185.22.6702-6706.2003 14594845 PMC262115

[16] Turtaut F, Lopez M, Ouahrani-Bettache S, Köhler S, Winum J-Y. 2014. Oxo- and thiooxo-imidazo[1,5-c]pyrimidine molecule library: beyond their interest in inhibition of Brucella suis histidinol dehydrogenase, a powerful protection tool in the synthesis of histidine analogues. Bioorg Med Chem Lett 24:5008–5010. doi:10.1016/j.bmcl.2014.09.020 25278235

[17] Abdo M-R, Joseph P, Mortier J, Turtaut F, Montero J-L, Masereel B, Köhler S, Winum J-Y. 2011. Anti-virulence strategy against Brucella suis: synthesis, biological evaluation and molecular modeling of selective histidinol dehydrogenase inhibitors. Org Biomol Chem 9:3681–3690. doi:10.1039/c1ob05149k 21461427

[18] Kohler S, Foulongne V, Ouahrani-Bettache S, Bourg G, Teyssier J, Ramuz M, Liautard J-P. 2002. The analysis of the intramacrophagic virulome of Brucella suis deciphers the environment encountered by the pathogen inside the macrophage host cell. Proc Natl Acad Sci USA 99:15711–15716. doi:10.1073/pnas.232454299 12438693 PMC137781

[19] Li T, Zhan Z, Lin Y, Lin M, Xie Q, Chen Y, He C, Tao J, Li C. 2019. Biosynthesis of amino acids in Xanthomonas oryzae pv. oryzae is essential to its pathogenicity. Microorganisms 7:693. doi:10.3390/microorganisms7120693 31847108 PMC6956189

[20] Li Z, Liu Y, Fu J, Zhang B, Cheng S, Wu M, Wang Z, Jiang J, Chang C, Liu X. 2019. Salmonella proteomic profiling during infection distinguishes the intracellular environment of host cells. mSystems 4:e00314-18. doi:10.1128/mSystems.00314-18 30984873 PMC6456673

[21] Dickey SW, Cheung GYC, Otto M. 2017. Different drugs for bad bugs: antivirulence strategies in the age of antibiotic resistance. Nat Rev Drug Discov 16:457–471. doi:10.1038/nrd.2017.23 28337021 PMC11849574

[22] Campbell MJ, Beenken KE, Ramirez AM, Smeltzer MS. 2024. Increased production of aureolysin and staphopain A is a primary determinant of the reduced virulence of Staphylococcus aureus sarA mutants in osteomyelitis. mBio 15:e0338323. doi:10.1128/mbio.03383-23 38415646 PMC11005355

[23] Nunes JES, Ducati RG, Breda A, Rosado LA, de Souza BM, Palma MS, Santos DS, Basso LA. 2011. Molecular, kinetic, thermodynamic, and structural analyses of Mycobacterium tuberculosis hisD-encoded metal-dependent dimeric histidinol dehydrogenase (EC 1.1.1.23). Arch Biochem Biophys 512:143–153. doi:10.1016/j.abb.2011.05.020 21672513

[24] Wang Y, Wang L, Zhang J, Duan X, Feng Y, Wang S, Shen L. 2020. PA0335, a gene encoding histidinol phosphate phosphatase, mediates histidine auxotrophy in Pseudomonas aeruginosa. Appl Environ Microbiol 86:e02593-19. doi:10.1128/AEM.02593-19 31862725 PMC7028973

[25] Xiao M, Zhao R, Zhang Q, Fan X, O’Sullivan MVN, Li D-F, Wang X-Y, Wu H-L, Kong F, Xu Y-C. 2016. Genotypic diversity of Staphylococcus aureus α-hemolysin gene (hla) and its association with clonal background: implications for vaccine development. PLoS One 11:e0149112. doi:10.1371/journal.pone.0149112 26866483 PMC4750931

[26] Koop G, Vrieling M, Storisteanu DML, Lok LSC, Monie T, van Wigcheren G, Raisen C, Ba X, Gleadall N, Hadjirin N, et al.. 2017. Identification of LukPQ, a novel, equid-adapted leukocidin of Staphylococcus aureus. Sci Rep 7:40660. doi:10.1038/srep40660 28106142 PMC5247767

[27] Raafat D, Otto M, Reppschläger K, Iqbal J, Holtfreter S. 2019. Fighting Staphylococcus aureus biofilms with monoclonal antibodies. Trends Microbiol 27:303–322. doi:10.1016/j.tim.2018.12.009 30665698 PMC6420399

[28] Wu S-C, Liu F, Zhu K, Shen J-Z. 2019. Natural products that target virulence factors in antibiotic-resistant Staphylococcus aureus. J Agric Food Chem 67:13195–13211. doi:10.1021/acs.jafc.9b05595 31702908

[29] Oliveira D, Borges A, Simões M. 2018. Staphylococcus aureus toxins and their molecular activity in infectious diseases. Toxins (Basel) 10:252. doi:10.3390/toxins10060252 29921792 PMC6024779

[30] The PLOS ONE Staff. 2015. Correction: L-histidine inhibits biofilm formation and FLO11-associated phenotypes in Saccharomyces cerevisiae flor yeasts. PLoS One 10:e0118167. doi:10.1371/journal.pone.0118167 25635823 PMC4311975

[31] James P, Marko-Varga GA. 2011. The international proteomics tutorial programme – reaching out to the next generation proteome scientists. J Proteome Res 10:3311–3312. doi:10.1021/pr200632u 21815689

[32] Audretsch C, Gratani F, Wolz C, Dandekar T. 2021. Modeling of stringent-response reflects nutrient stress induced growth impairment and essential amino acids in different Staphylococcus aureus mutants. Sci Rep 11:9651. doi:10.1038/s41598-021-88646-1 33958641 PMC8102509

[33] Ding W, Zhou Y, Qu Q, Cui W, God’spower BO, Liu Y, Chen X, Chen M, Yang Y, Li Y. 2018. Azithromycin inhibits biofilm formation by Staphylococcus xylosus and affects histidine biosynthesis pathway. Front Pharmacol 9:740. doi:10.3389/fphar.2018.00740 30042679 PMC6048454

[34] Vudhya Gowrisankar Y, Manne Mudhu S, Pasupuleti SK, Suthi S, Chaudhury A, Sarma P. 2021. Staphylococcus aureus grown in anaerobic conditions exhibits elevated glutamine biosynthesis and biofilm units. Can J Microbiol 67:323–331. doi:10.1139/cjm-2020-0434 33136443

[35] Kimura T, Kobayashi K. 2020. Role of glutamate synthase in biofilm formation by bacillus subtilis. J Bacteriol 202:e00120-20. doi:10.1128/JB.00120-20 PMC7317036 32393519

[36] Matsumoto M, Nakagawa S, Zhang L, Nakamura Y, Villaruz AE, Otto M, Wolz C, Inohara N, Núñez G. 2021. Interaction between Staphylococcus Agr virulence and neutrophils regulates pathogen expansion in the skin. Cell Host Microbe 29:930–940. doi:10.1016/j.chom.2021.03.007 33852876 PMC11024063

[37] Li M, Wang B, Chen J, Jiang L, Zhou Y, Guo G, Jiang F, Hu Y, Wang C, Yang Y, Tang J, Han P, Yu J, Shen H. 2024. Staphylococcus aureus SaeRS impairs macrophage immune functions through bacterial clumps formation in the early stage of infection. NPJ Biofilms Microbiomes 10:102. doi:10.1038/s41522-024-00576-8 39370453 PMC11456606

[38] Kwiecinski JM, Kratofil RM, Parlet CP, Surewaard BGJ, Kubes P, Horswill AR. 2021. Staphylococcus aureus uses the ArlRS and MgrA cascade to regulate immune evasion during skin infection. Cell Rep 36:109462. doi:10.1016/j.celrep.2021.109462 34320352 PMC8450000

[39] Rao Y, Peng H, Shang W, Hu Z, Yang Y, Tan L, Li M, Zhou R, Rao X. 2022. A vancomycin resistance-associated WalK(S221P) mutation attenuates the virulence of vancomycin-intermediate Staphylococcus aureus. J Adv Res 40:167–178. doi:10.1016/j.jare.2021.11.015 36100324 PMC9481939

[40] Lu Y, Chen F, Zhao Q, Cao Q, Chen R, Pan H, Wang Y, Huang H, Huang R, Liu Q, Li M, Bae T, Liang H, Lan L. 2023. Modulation of MRSA virulence gene expression by the wall teichoic acid enzyme TarO. Nat Commun 14:1594. doi:10.1038/s41467-023-37310-5 36949052 PMC10032271

[41] Cho J, Costa SK, Wierzbicki RM, Rigby WFC, Cheung AL. 2021. The extracellular loop of the membrane permease VraG interacts with GraS to sense cationic antimicrobial peptides in Staphylococcus aureus. PLoS Pathog 17:e1009338. doi:10.1371/journal.ppat.1009338 33647048 PMC7951975

[42] Dmitriev A, Chen X, Paluscio E, Stephens AC, Banerjee SK, Vitko NP, Richardson AR. 2021. The intersection of the Staphylococcus aureus Rex and SrrAB regulons: an example of metabolic evolution that maximizes resistance to immune radicals. mBio 12:e0218821. doi:10.1128/mBio.02188-21 34781744 PMC8593685

[43] Tang C, Li J, Shen Y, Liu M, Liu H, Liu H, Xun L, Xia Y. 2023. A sulfide-sensor and a sulfane sulfur-sensor collectively regulate sulfur-oxidation for feather degradation by Bacillus licheniformis. Commun Biol 6:167. doi:10.1038/s42003-023-04538-2 36765168 PMC9918477

[44] Pi H, Carlin SM, Beavers WN, Hillebrand GH, Krystofiak ES, Stauff DL, Skaar EP. 2024. FapR regulates HssRS-mediated heme homeostasis in Bacillus anthracis. bioRxiv:2024.07.08.602573. doi:10.1101/2024.07.08.602573 PMC12153329 40407322

[45] Endres JL, Chaudhari SS, Zhang X, Prahlad J, Wang S-Q, Foley LA, Luca S, Bose JL, Thomas VC, Bayles KW. 2021. The Staphylococcus aureus CidA and LrgA proteins are functional holins involved in the transport of by-products of carbohydrate metabolism. mBio 13:e0282721. doi:10.1128/mbio.02827-21 35100878 PMC8805020

[46] Liu Q, Yeo W-S, Bae T. 2016. The SaeRS two-component system of Staphylococcus aureus. Genes (Basel) 7:81. doi:10.3390/genes7100081 27706107 PMC5083920

[47] Mizar P, Arya R, Kim T, Cha S, Ryu K-S, Yeo W-S, Bae T, Kim DW, Park KH, Kim KK, Lee SS. 2018. Total synthesis of xanthoangelol B and its various fragments: toward inhibition of virulence factor production of Staphylococcus aureus. J Med Chem 61:10473–10487. doi:10.1021/acs.jmedchem.8b01012 30388007 PMC6326535

[48] Ghosh M, Wang LC, Huber RG, Gao Y, Morgan LK, Tulsian NK, Bond PJ, Kenney LJ, Anand GS. 2019. Engineering an osmosensor by pivotal histidine positioning within disordered helices. Structure 27:302–314. doi:10.1016/j.str.2018.10.012 30503779 PMC6377431

[49] Poudel S, Hefner Y, Szubin R, Sastry A, Gao Y, Nizet V, Palsson BO. 2022. Coordination of CcpA and CodY regulators in Staphylococcus aureus USA300 strains. mSystems 7:e0048022. doi:10.1128/msystems.00480-22 36321827 PMC9765215

[50] Mlynek KD, Sause WE, Moormeier DE, Sadykov MR, Hill KR, Torres VJ, Bayles KW, Brinsmade SR. 2018. Nutritional regulation of the sae two-component system by CodY in Staphylococcus aureus. J Bacteriol 200:e00012-18. doi:10.1128/JB.00012-18 29378891 PMC5869476

[51] Hasinoff BB, Wu X, Patel D, Kanagasabai R, Karmahapatra S, Yalowich JC. 2016. Mechanisms of action and reduced cardiotoxicity of pixantrone; a topoisomerase II targeting agent with cellular selectivity for the topoisomerase IIα isoform. J Pharmacol Exp Ther 356:397–409. doi:10.1124/jpet.115.228650 26660439 PMC4746493

[52] Barrenetxea Lekue C, Grasso Cicala S, Leppä S, Stauffer Larsen T, Herráez Rodríguez S, Alonso Caballero C, Jørgensen JM, Toldbod H, Leal Martínez I, D’Amore F. 2019. Pixantrone beyond monotherapy: a review. Ann Hematol 98:2025–2033. doi:10.1007/s00277-019-03749-0 31312929 PMC6700039

[53] Evison BJ, Mansour OC, Menta E, Phillips DR, Cutts SM. 2007. Pixantrone can be activated by formaldehyde to generate a potent DNA adduct forming agent. Nucleic Acids Res 35:3581–3589. doi:10.1093/nar/gkm285 17483512 PMC1920253

[54] Cavalletti E, Crippa L, Mainardi P, Oggioni N, Cavagnoli R, Bellini O, Sala F. 2007. Pixantrone (BBR 2778) has reduced cardiotoxic potential in mice pretreated with doxorubicin: comparative studies against doxorubicin and mitoxantrone. Invest New Drugs 25:187–195. doi:10.1007/s10637-007-9037-8 17285358

[55] Longo M, Della Torre P, Allievi C, Morisetti A, Al-Fayoumi S, Singer JW. 2014. Tolerability and toxicological profile of pixantrone (Pixuvri) in juvenile mice. Comparative study with doxorubicin. Reprod Toxicol 46:20–30. doi:10.1016/j.reprotox.2014.02.006 24602559

[56] Cavaletti G, Cavalletti E, Crippa L, Di Luccio E, Oggioni N, Mazzanti B, Biagioli T, Sala F, Sala V, Frigo M, Rota S, Tagliabue E, Stanzani L, Galbiati S, Rigolio R, Zoia C, Tredici G, Perseghin P, Dassi M, Riccio P, Lolli F. 2004. Pixantrone (BBR2778) reduces the severity of experimental allergic encephalomyelitis. J Neuroimmunol 151:55–65. doi:10.1016/j.jneuroim.2004.02.008 15145604

[57] Minotti G, Han H, Cattan V, Egorov A, Bertoni F. 2018. Pixantrone: novel mode of action and clinical readouts. Expert Rev Hematol 11:587–596. doi:10.1080/17474086.2018.1476848 29912583

[58] She P, Li Z, Li Y, Liu S, Li L, Yang Y, Zhou L, Wu Y. 2022. Pixantrone sensitizes Gram-negative pathogens to rifampin. Microbiol Spectr 10:e0211422. doi:10.1128/spectrum.02114-22 36318018 PMC9769682

[59] Peng Q, Guo L, Dong Y, Bao T, Wang H, Xu T, Zhang Y, Han J. 2022. PurN is involved in antibiotic tolerance and virulence in Staphylococcus aureus. Antibiotics (Basel) 11:1702. doi:10.3390/antibiotics11121702 36551359 PMC9774800

[60] Zhao F, Yan H, Zheng Y, Zu Y, Yang S, Hu H, Shi S, Liang H, Niu X. 2023. Joint concanavalin A-aptamer enabled dual recognition for anti-interference visual detection of Salmonella typhimurium in complex food matrices. Food Chem 426:136581. doi:10.1016/j.foodchem.2023.136581 37311299

[61] Xu T, Wang X-Y, Cui P, Zhang Y-M, Zhang W-H, Zhang Y. 2017. The Agr quorum sensing system represses persister formation through regulation of phenol soluble modulins in Staphylococcus aureus. Front Microbiol 8:2189. doi:10.3389/fmicb.2017.02189 29163457 PMC5681930

[62] Zheng Y, Shang W, Peng H, Rao Y, Zhao X, Hu Z, Yang Y, Hu Q, Tan L, Xiong K, Li S, Zhu J, Hu X, Zhou R, Li M, Rao X. 2019. Virulence determinants are required for brain abscess formation through Staphylococcus aureus infection and are potential targets of antivirulence factor therapy. Front Microbiol 10:682. doi:10.3389/fmicb.2019.00682 31024479 PMC6460967

[63] Morata-Moreno N, Pérez-Tanoira R, Del Campo-Balguerias A, Carrillo-Hermosilla F, Hernando-Gozalo M, Rescalvo-Casas C, Ocana AV, Segui P, Alonso-Moreno C, Pérez-Martínez FC, Molina-Alarcón M. 2024. A new guanidine-core small-molecule compound as a potential antimicrobial agent against resistant bacterial strains. Antibiotics (Basel) 13:609. doi:10.3390/antibiotics13070609 39061291 PMC11274109

[64] Song K-H, Kim M, Kim CJ, Cho JE, Choi YJ, Park JS, Ahn S, Jang H-C, Park K-H, Jung S-I, Yoon N, Kim D-M, Hwang J-H, Lee CS, Lee JH, Kwak YG, Kim ES, Park SY, Park Y, Lee KS, Lee Y-S, Kim HB. 2017. Impact of vancomycin MIC on treatment outcomes in invasive Staphylococcus aureus infections. Antimicrob Agents Chemother 61:e01845-16. doi:10.1128/AAC.01845-16 27956430 PMC5328555

[65] Borman AM, Muller J, Walsh-Quantick J, Szekely A, Patterson Z, Palmer MD, Fraser M, Johnson EM. 2020. MIC distributions for amphotericin B, fluconazole, itraconazole, voriconazole, flucytosine and anidulafungin and 35 uncommon pathogenic yeast species from the UK determined using the CLSI broth microdilution method. J Antimicrob Chemother 75:1194–1205. doi:10.1093/jac/dkz568 32025716

[66] Hartmann G, Lopes CE, de Dos Reis Paula A, Paz MC, Tres GZ, Silva VGC, de Moraes JTR, Araújo MD, Machado REH, Terra JA, Sonne L. 2025. Septicemic omphalophlebitis by Streptococcus equi subsp. zooepidemicus in a southern right whale calf (Eubalaena australis). Vet Res Commun 49:84. doi:10.1007/s11259-025-10650-x 39826022

[67] Rao L, Xu Y, Shen L, Wang X, Zhao H, Wang B, Zhang J, Xiao Y, Guo Y, Sheng Y, Cheng L, Song Z, Yu F. 2022. Small-molecule compound SYG-180-2-2 attenuates Staphylococcus aureus virulence by inhibiting hemolysin and staphyloxanthin production. Front Cell Infect Microbiol 12:1008289. doi:10.3389/fcimb.2022.1008289 36310881 PMC9606476

[68] Du C, Si Y, Pang N, Li Y, Guo Y, Liu C, Fan H. 2022. Prokaryotic expression, purification, physicochemical properties and antifungal activity analysis of phloem protein PP2-A1 from cucumber. Int J Biol Macromol 194:395–401. doi:10.1016/j.ijbiomac.2021.11.081 34822821

[69] Ortiz-Riaño EJ, Mancera-Zapata DL, Ulloa-Ramírez M, Arce-Vega F, Morales-Narváez E. 2022. Measurement of protein kinetics using a liquid phase-based biosensing platform. Anal Chem 94:15553–15557. doi:10.1021/acs.analchem.2c03305 36253365

