# Supplementary material for: Histidinol dehydrogenase (HisD): a critical regulator of Staphylococcus aureus virulence and a promising target for antivirulence therapy
Source: Microbiol Spectr. 2025 Nov 26;14(1):e01429-25. doi: 10.1128/spectrum.01429-25 (PMC12772333; doi:10.1128/spectrum.01429-25)
Supplement: Supplemental material — Fig. S1; Tables S1 to S3. [file spectrum.01429-25-s0001.docx]

**Supplementary materials Table. S1**

Table. S1 Strains and plasmids used in this study

| **Strains or Plasmids** | **Features and Uses** | **Source** |
| --- | --- | --- |
| *S. aureus* |  |  |
| Newman | ATCC25904, Clinical MSSA isolates | ATCC |
| Δ*hisD* | The Newman *hisD* deletion strain | this study |
| Δ*hisD*::pRAB11 | pRAB11 electric transfer into Δ*hisD* | this study |
| Δ*hisD*::pRAB-*hisD* | pRAB-*hisD* electric transfer into Δ*hisD* | this study |
| WT::pRAB11 | pRAB11 electric transfer into Newman | this study |
| WT::pRAB-*hisD* | pRAB-*hisD* electric transfer into Newman | this study |
| ATCC25923 | Used to evaluate the impact of pixantrone measuring the bacterial hemolysis rate and the expression of virulence factor genes upon intervention. | ATCC |
| ATCC29213 |  | ATCC |
| Clinical strains MSSA（3 strains） |  | Lanzhou University Second Hospital，China |
| Clinical strains MRSA（7 strains） |  |  |
| *E. coli* |  |  |
| DH5α | The *E. coli* DH5α cloning strain | (1) |
| DC10B | The *E. coli* DC10B cloning strain | (2) |
| BL21 | The *E. coli* BL21 cloning strain | Lanzhou University, China |
| Plasmids |  |  |
| pBT2 | The shuttle plasmids from *E. coli* and *S. aureus* are specifically designed for gene knockout and provide resistance to ampicillin and chloramphenicol. | (3) |
| pRAB11 | The shuttle plasmids from *E. coli* and *S. aureus* are specifically designed for gene complementation, providing resistance to ampicillin and chloramphenicol, and include a promoter that is inducible by tetracycline. | (4) |
| pRAB-*hisD* | The *hisD* gene was inserted into the pRAB11 plasmid to achieve gene complementation and overexpression. | this study |
| pET30a | Prokaryotic protein expression vector, used for high-level expression of recombinant protein in *E. coli.* | Lanzhou University, China |
| pET30a-*hisD* | The *hisD* gene was inserted into the pET30a plasmid to achieve Protein expression. | this study |

1. Niu H, Hu L, Li Q, Da Z, Wang B, Tang K, Xin Q, Yu H, Zhang Y, Wang Y, Ma X, Zhu B. 2011. Construction and evaluation of a multistage mycobacterium tuberculosis subunit vaccine candidate Mtb10.4-HspX. Vaccine 29:9451–9458.

2. Xu T, Wang X-Y, Cui P, Zhang Y-M, Zhang W-H, Zhang Y. 2017. The agr quorum sensing system represses persister formation through regulation of phenol soluble modulins in staphylococcus aureus. Front Microbiol 8:2189.

3. Yang Y, Tan L, He S, Hao B, Huang X, Zhou Y, Shang W, Peng H, Hu Z, Ding R, Rao X. 2024. Sub-MIC vancomycin enhances the antibiotic tolerance of vancomycin-intermediate staphylococcus aureus through downregulation of protein succinylation. Microbiol Res 282:127635.

4. Peng Q, Guo L, Dong Y, Bao T, Wang H, Xu T, Zhang Y, Han J. 2022. PurN is involved in antibiotic tolerance and virulence in staphylococcus aureus. Antibiotics (Basel) 11:1702.

**Supplementary materials Table. S2**

Table. S2 The primers used for *hisD* knockout, complement and overexpression

| Gene | sequence (5’→3’) |
| --- | --- |
| *hisD*-uf | ATGCCTGCAGGTCGACTAAAAGCGAGGTTTAAATCCTTGTTCATATACGTATTG |
| *hisD*-ur | TATTGCCAGGGAGTGTAATTGAAATGATTTATATTGATAAAAATGAAAGTCC |
| *hisD*-df | CACTCCCTGGCAATAGACACCTCCAAAGAGC |
| *hisD*-dr | CGGTACCCGGGGATCCTCCAATCGTGCAAATCTTGAAAGAGAATACG |
| *hisD*-f | TGATAGAGTATGATGGTACCCTAAGACTGACGTATTAAAATAGACTGCTGGT |
| *hisD*-r | GACGGCCAGTGAATTCATGCTTAATGCACAACAATTTTTAAATCAATTTTCATTAGAAG |

**Supplementary materials Table. S3**

Table. S3 The primers used for qRT-PCR

| Gene | sequence (5’→3’) |
| --- | --- |
| *coa*-F | ATGAAAAAGCAAATAATTTCGCTAGGCG |
| *coa*-R | TTATTTTGTTACTCTAGGCCCATATGTCGC |
| *hlgA*-F | TCCAATCAGCGCCATCAA |
| *hlgA*-R | ACACCTTTAGAGTTCTGACTTTCT |
| *hlgB*-F | AATGTTGGCTGGGGAGT |
| *hlgB*-R | CGCTATGAAGTTTTGGC |
| *hlgC*-F | CGCCACTGAATCAGGTCAAA |
| *hlgC*-R | ACTCACTGTCTGGAACGAAATAA |
| *lukS*-F | GACCAGTGTACATGCCAGTTATT |
| *lukS*-R | AGCTCAGGTGGTAAATTCGATTC |
| *lukF*-F | TCAGACACAGTTACAGGCA |
| *lukF*-R | GTTGGAAAGTAGAAGCACA |
| *lukD*-F | GGTGCATAGTCAACAACATTTACA |
| *lukD*-R | GGCAGCCGGAAACATTAATTC |
| *lukE*-F | AGATGGTGCTGACTGGAAATTA |
| *lukE*-R | GGACTGACGACTAAAGATCCAAA |
| *hla*-F | GCTTTGTTAGGATCAAGGAAGTTATC |
| *hla*-R | AGATTCTTGGAACCCGGTATATG |
| *eta*-F | GTCGATGTGTTCGGTTTGATTG |
| *eta*-R | GCACCCGTTAGCGGATATT |
| *sea*-F | CTAAAGCTGCTCCCTGCAAT |
| *sea*-R | CCCTAACGTGGACAACAAGTC |
| *NWMN＿1873*-F | AGTTGCAACACTTGCATTAGC |
| *NWMN＿1873*-R | TTAGATTCGGCTTTGGCACTAT |
| *NWMN＿1926*-F | TTGTGGTGCTGGACATGAT |
| *NWMN＿1926*-R | TGAACTCTGGAGTGCCTTTATT |
| *NWMN＿2071*-F | GGTTAACTTTGACAGCTATGGATTT |
| *NWMN＿2071*-R | CAGTTGTAGGTGGTTGGATAGG |
| *NWMN＿1503*-F | CTATTTCTGTTTGATGTCCG |
| *NWMN＿1503*-R | GCAATGTAGTTATGGTGGTG |
| *16S*-F | CGTGCTACAATGGACAATACAAA |
| *16S*-R | ATCTACGATTACTAGCGATTCCA |
| *hlb*-F | CGTAGCGATTGTAAGTAA |
| *hlb*-R | TCTTCAGATTGTGTATGTG |
| *saeR*-F | GTCGTAACCATTAACTTCTG |
| *saeR*-R | ATCGTGGATGATGAACAA |
| *saeS*-F | CGTTCTTGTAGTTCTGGTAT |
| *saeS*-R | GTTGGTAGTCGCATTGATA |
| *hisD*-F | CATTAGGTTGAGGTGGTG |
| *hisD*-R | TGTCGGTATTTATGTGCC |
| *agrA-*F | GCCTATGGAAATTGCCCTCG |
| *agrA-*R | TTAGC TTGCTCAAGCACCTC |
| *agrC-*F | TGATAGCGCGTCCTTAATC |
| *agrC-*R | GCACAGTATAGAATAATCTGCG |
| *NWMN＿1327-*F | GCAATAGATAATCCGAGTCCA |
| *NWMN＿1327-*R | CAGATCATGGAATTGGTATTCC |
| *NWMN＿1328-*F | GCCACGAACTGTTTCAATCA |
| *NWMN＿1328-*R | AAACCATGTTATGCAACGGG |
| *NWMN＿0018-*F | GCAGTACGCGAAACAATTAG |
| *NWMN＿0018-*R | CCAATTTCTTGACGGTTGGC |
| *NWMN＿0939-*F | CATGCTGGTTATGAAGCTGT |
| *NWMN＿0939-*R | GGCGCTGTCATATTTAATTGTG |
| *NWMN＿2263-*F | GGTGCAATGTCTTGTTGTCG |
| *NWMN＿2263-*R | GGTGCAATGTCTTGTTGTCG |
| *NWMN＿2264-*F | AGTCCACAAGCTGAAGCAC |
| *NWMN＿2264-*R | GGATTGTACCATGATGCAATTC |
| *NWMN＿2291-*F | GCTCAGGTTTACTCTTTAAGC |
| *NWMN＿2291-*R | TTGCCACTTATTGCCAAAGG |
| *srrA-*F | CGGTGTTTGTTTATGTTCACG |
| *srrA-*R | GGCTTATGAACTTGCAATGG |
| *srrB-*F | CGGTTCTGTAACAATACCATC |
| *srrB-*R | CGTGATATGACTAATGAGCAC |
| *lytS-*F | AGTTGGTGGCCCTTTTGTAG |
| *lytS-*R | CTTGTAAGCCAAAATAACCAGC |
| *NWMN＿0017-*F | CGTCGTCATTACTCACAACC |
| *NWMN＿0017-*R | ATTCAATATCTTCGCCACG |
| *aur-*F | TGTCGGCTGCGTCATCTTTA |
| *aur-*R | GCAACCGAGTGTTGATGGTG |
| *scpA-*F | CGCCCAACTAAATCTTCCCG |
| *scpA-*R | ACGTTCATGCAATGAAACAGCTT |
| *sspA-*F | TGCGTAGCATCTACGACGTG |
| *sspA-*R | AACGCAGTCAAGCAAACAGC |
| *sspB-*F | GCTGCCATACTGAATCCTGC |
| *sspB-*R | AAAGCTACGCCTCTACCTGG |

**Supplementary materials Fig. S1**


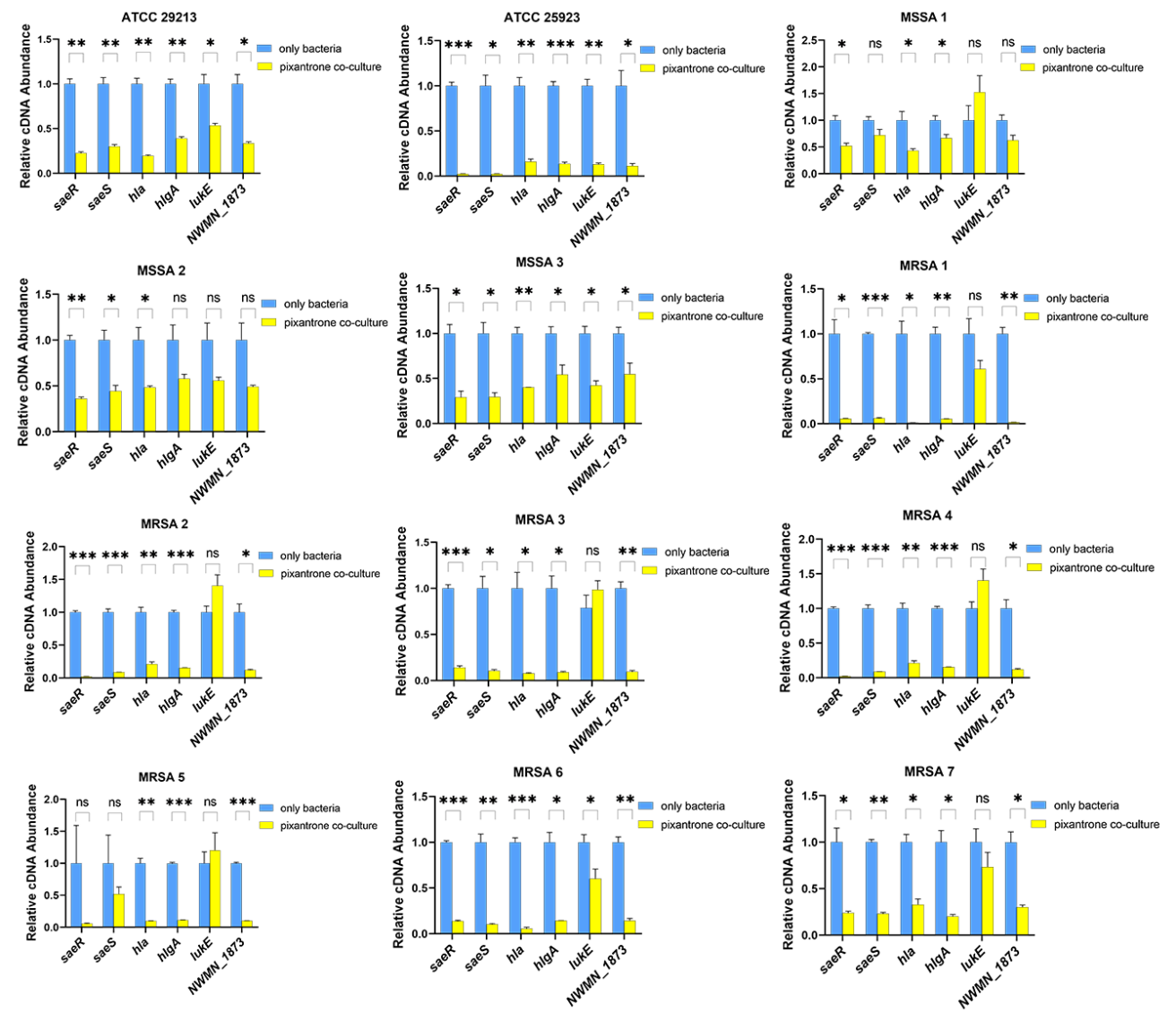


**Fig. S1 Pixantrone modulates SaeRS regulon gene and virulence gene expression in clinical *S. aureus* isolates.** Pixantrone (200μM) treatment (co-culture with strains in TSB 24h ) significantly decreased the expression of most virulence-related genes (*p* < 0.05). The following genes were not significantly affected: *lukE* ((MSSA 1 and 2, MRSA 1, 2, 3, 4, 5 and 7)), *saeR* (MRSA 5), *saeS* (MSSA1, MRSA5), *NWMN_1873* (MSSA1, MSSA2), and *hlgA* (MSSA2). **P* < 0.05, ***P* < 0.01, ****P* < 0.001, ns *P* > 0.05.
